# Ferritin‐Based Supramolecular Assembly Drug Delivery System for Aminated Fullerene Derivatives to Enhance Tumor‐Targeted Therapy

**DOI:** 10.1002/advs.202413389

**Published:** 2024-12-31

**Authors:** Baoli Zhang, Libin Yang, Yiliang Jin, Yicheng Lu, Jianru Li, Guoheng Tang, Yang Liu, Jiawei Huo, Ran Xu, Chunru Wang, Xiyun Yan, Jie Li, Kelong Fan

**Affiliations:** ^1^ Zhejiang Provincial Key Laboratory of Pancreatic Disease the First Affiliated Hospital Zhejiang University School of Medicine Hangzhou 310003 P. R. China; ^2^ CAS Engineering Laboratory for Nanozyme Key Laboratory of Protein and Peptide Pharmaceutical Institute of Biophysics Chinese Academy of Sciences Beijing 100101 P. R. China; ^3^ Beijing National Research Center for Molecular Sciences Key Laboratory of Molecular Nanostructure and Nanotechnology Institute of Chemistry Chinese Academy of Science Beijing 100190 P. R. China; ^4^ University of Chinese Academy of Sciences Chinese Academy of Sciences Beijing 100408 P. R. China; ^5^ Nanozyme Laboratory in Zhongyuan Henan Academy of Innovations in Medical Science Zhengzhou Henan 451163 P. R. China

**Keywords:** aminated fullerene derivative, drug delivery system, ferritin, supramolecular assembly, tumor‐targeted therapy

## Abstract

Owing to their attractive antitumor effects, aminated fullerene derivatives are emerging as promising therapeutic drugs for cancer. However, their in vivo applications are severely limited due to cation toxicity. To address this problem, human heavy chain ferritin (HFn), possessing natural biocompatibility is utilized, to develop a novel supramolecular assembly drug delivery system. Specifically, tetra[4‐(amino)piperidin‐1‐yl]‐C_60_ (TAPC) is selected as the representative aminated fullerene, and a layer‐by‐layer assembly strategy is designed to controllably assemble TAPC with the negatively charged HFn into a hierarchical coassembly (H@T@H) via electrostatic interactions and hydrogen bonds. In this ordered multilayer structure, the surface displayed HFn endows the inner TAPC with biocompatibility, tumor‐targeting and blood‐brain barrier crossing ability. Additionally, the electrostatic assembly mode enables the acid‐responsive disassembly of H@T@H to release TAPC in lysosomes. In the orthotopic glioma mouse model, the HFn‐assembled TAPC (H@T@H) shows higher brain accumulation and a stronger inhibitory effect on glioma than polyethylene glycol (PEG)‐coated TAPC. Moreover, in an experimental metastasis mouse model, H@T@H have significant preventive and therapeutic effects on tumor metastasis. Encouragingly, the ferritin‐based supramolecular assembly strategy has been proven to have broad applicability for various aminated fullerene derivatives, showing promising potential for tackling the in vivo delivery challenges of cationic drugs.

## Graphical abstract

This study utilizes the naturally negatively charged ferritin for supramolecular assembly with aminated fullerene derivatives through electrostatic interactions and hydrogen bonds. Leveraging an ordered layer‐by‐layer structure, the novel ferritin‐based drug delivery system effectively addresses the cation toxicity concerns of aminated fullerenes and endows them with tumor‐targeting ability, paving the pathway for their in vivo biomedical application in cancer therapy.

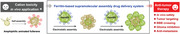

## 1 Introduction

Fullerene derivatives are currently emerging as potential antitumor molecules and have attracted widespread attention in the field of cancer therapy.^[^ ^1^, ^2^, ^3^ ^]^ Fullerenes are a group of carbon‐based cage‐like molecules with strong hydrophobicity. To improve water solubility, a series of fullerene derivatives have been synthesized by chemically coupling hydrophilic groups (e.g., hydroxyl, amino, and carboxyl groups) on the surface of fullerene nanocages.^[^ ^4^, ^5^, ^6^, ^7^, ^8^ ^]^ Notably, these fullerene derivatives have been shown to possess significant inhibitory activities against many types of cancers via diverse mechanisms.^[^ ^9^, ^10^, ^11^ ^]^ Among these water‐soluble fullerene derivatives, aminated fullerenes exhibit prominent antitumor activity, which can trigger G0/G1 cell cycle arrest, reverse epithelial‐mesenchymal transition (EMT) and reduce the metastatic potential of tumor cells, showing great potential for development as potent antitumor medicines.^[^ ^7^, ^12^, ^13^, ^14^ ^]^ However, the safety concerns of cation toxicity caused by positively charged amino modifications severely hinder the in vivo application and clinical translation of aminated fullerene derivatives. The surface cation property of nanoparticles is very likely to bind non‐specifically to the negatively charged membranes of normal cells and serum proteins in vivo, leading to acute cell damage and harmful immune reactions.^[^ ^15^, ^16^, ^17^, ^18^ ^]^ Therefore, aminated fullerene derivatives are in urgent need of suitable drug delivery systems to address their in vivo toxicity issue.

Supramolecular assembly driven by electrostatic interactions provides a new idea for constructing a delivery system for charged drugs. Studies have proven that oppositely charged nanoparticles and/or molecules can self‐assemble and form highly stable coassemblies.^[^ ^19^, ^20^, ^21^ ^]^ Notably, by taking advantage of the layer‐by‐layer (LBL) assembly technique, drugs can realize surface modification to optimize properties, improve biocompatibility, reduce off‐target effects, and enhance therapeutic efficacy.^[^ ^22^, ^23^, ^24^, ^25^ ^]^ In light of this, supramolecular assembly is a promising and feasible mean to endow aminated fullerenes with biocompatible properties. Ferritin, a naturally negatively charged protein widely applied as a drug carrier, is a favorable candidate for the electrostatic assembly with aminated fullerenes.^[^ ^26^, ^27^ ^]^ Compared with chemically synthesized materials, which have the drawbacks of poor biodegradability and potential toxicity, ferritin possesses numerous advantages. First, as an endogenous protein, ferritin exhibits excellent biological properties, such as low immunogenicity and good biocompatibility. Second, ferritin has an inherent tumor‐targeting ability, which can specifically recognize and bind to tumor cells via transferrin receptor 1 (TfR1).^[^ ^28^ ^]^ Third, ferritin can traverse the blood‐brain barrier (BBB), paving the way for brain tumor therapy.^[^ ^29^ ^]^ Most importantly, abundant amino acid residues exist on the surface of ferritin that provide potential interactions (e.g., electrostatic interactions and hydrogen bonds) for supramolecular assembly with aminated fullerenes. Given the above advantages, we selected human heavy chain ferritin (HFn) as the module to construct a supramolecular assembly drug delivery system for aminated fullerene derivatives.

**Figure 1:**
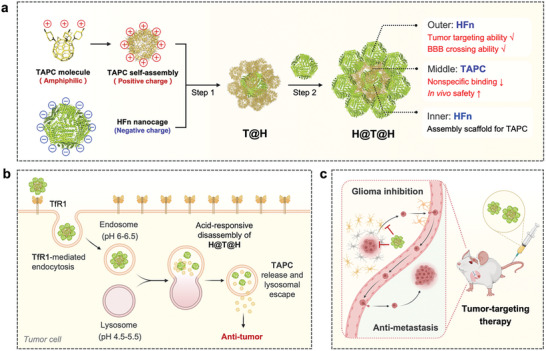
Schematic illustration of the ferritin‐based supramolecular assembly drug delivery system and its antitumor application. a) Construction of H@T@H via a simple two‐step method of LBL supramolecular assembly and the advantageous properties of the ordered multilayer structure. b) Relying on the outer layer of HFn, H@T@H can specifically recognize TfR1, the abnormally overexpressed receptor on tumor cells, to mediate endocytosis. After entering the cells, the acidic environment of lysosomes triggers the disassembly of H@T@H to release TAPC. c) H@T@H possesses tumor‐targeting and BBB‐crossing abilities and can effectively inhibit glioma progression and distant metastasis. The illustration was created with BioRender.com.

In this study, the LBL supramolecular assembly strategy was employed to assemble HFn and TAPC into a multilayer nanosystem with a sandwich structure, which we named H@T@H. In this hierarchical supramolecular coassembly, the external display of HFn not only endows the inner TAPC with biocompatibility to ensure in vivo safety, but also enables TAPC to cross the BBB and specifically accumulate in brain tumors. Compared with polyethylene glycol (PEG)‐coated TAPC, which lacks tumor‐targeting ability, the HFn‐based supramolecular system significantly enhanced the antitumor activity of TAPC both in vitro and in vivo. In the orthotopic glioma‐bearing mice, H@T@H specifically targeted brain tumors and significantly inhibited the growth of glioma. In the experimental metastasis model, H@T@H showed excellent therapeutic and preventive effects on tumor metastasis. Notably, the ferritin‐based supramolecular assembly strategy could be widely applied to various aminated fullerene derivatives, highlighting its universal application potential (**Figure** **1**).

## 2 Results and Discussion

### 2.1 Self‐Assembly of Amphiphilic TAPC Molecules

**Figure 2:**
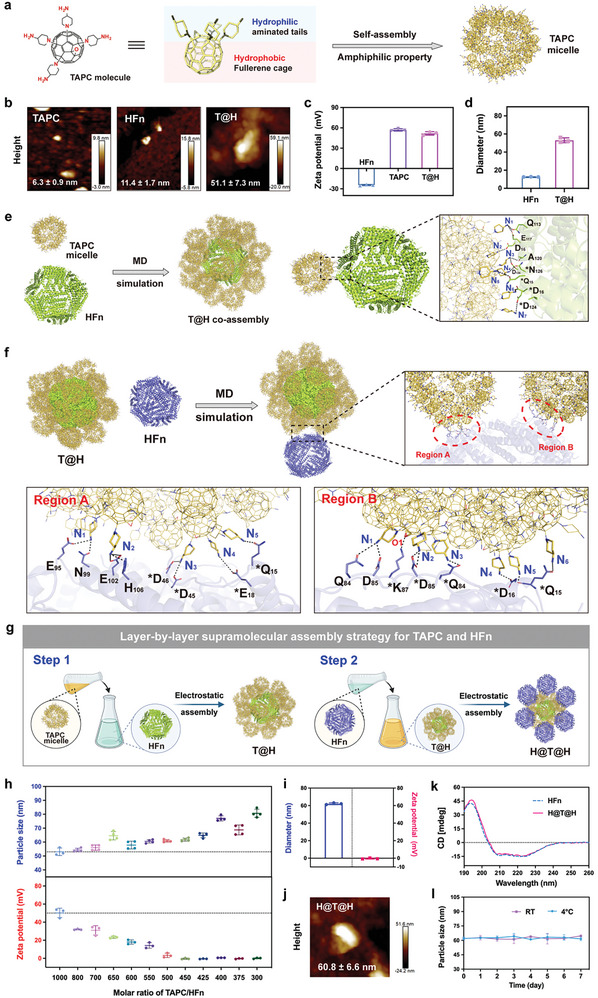
Investigation and evaluation of LBL supramolecular assembly between TAPC and HFn. a) Schematic diagram of the self‐assembly of amphiphilic TAPC molecules. b) Height signal maps of the HFn nanocage, TAPC micelle, and T@H coassembly were acquired from AFM. c) Characterization of the zeta potential (*n* = 4). d) Characterization of the particle size (*n* = 3). e) The final stable structure of the TAPC‐HFn coassembly obtained from the MD simulation (left). Analysis of the molecular binding modes between HFn nanocages and TAPC micelles (right). f) The final stable structure of the LBL assembly obtained from the MD simulation (above). Analysis of the molecular binding modes between HFn and T@H coassembly (below). As ferritin is a 24‐subunit protein, the two different subunits involved in the assembly are distinguished by asterisks. Abbreviations: Glutamic acid (Glu, E), Aspartic acid (Asp, D), Asparagine (Asn, N), Glutamine (Gln, Q), Alanine (Ala, A), Lysine (Lys, K), Histidine (His, H). g) Scheme of the two‐step method for LBL assembly of HFn‐TAPC. h) Investigating the changes in the particle size and zeta potential of the LBL assembly during the process of adding different amounts of HFn to T@H (*n* = 3–4). i) Hydrodynamic size and surface potential of H@T@H (*n* = 3). j) Height signal map of H@T@H acquired from AFM. k) CD spectra of HFn and H@T@H. l) Storage stability evaluation of H@T@H in solution. The H@T@H samples were placed at 4 ℃ or at room temperature (RT) for 7 days, and their particle sizes were detected by DLS (*n* = 3). All data are presented as mean ± SD.

Our previous studies successfully synthesized TAPC, an aminated [60]fullerene derivative with well‐defined structures (Figure S1, Supporting Information).^[^ ^14^ ^]^ Since only one end of the fullerene cage is modified with amino groups, TAPC molecules exhibit amphiphilic properties that mediate self‐assembly in aqueous solution (**Figure** **2**a). According to the molecular dynamics (MD) simulations (Figure S2a,b, Supporting Information), the TAPC molecules could spontaneously form a micelle‐like structure with a diameter of ≈61 Å (6.1 nm), which was consistent with the experimental results determined by atomic force microscopy (AFM) (Figure 2b). The MD results also revealed that the stable binding between TAPC molecules is mediated by the *π*–*π* stacking of hexagonal rings in the fullerene cage, with a binding energy of −6.18 kcal mol^−1^. Owing to steric hindrance, the hydrophilic aminated tails of adjacent TAPCs are staggered in different directions. Notably, the most aminated tails are oriented outward from the micelle structure, and only a few point inward (Figure S2c,d, Supporting Information). This external arrangement of numerous amino groups contributes to the positive charges of TAPC micelles.

### 2.2 Investigating the HFn‐Based Supramolecular Assembly of T@H

Related studies have demonstrated that nanoparticles with opposite charges can spontaneously form stable core‐shell aggregates via electrostatic interactions in an ion‐like behavior.^[^ ^19^, ^20^, ^21^ ^]^ Surface potential characterization confirmed the opposite charges of HFn and TAPC in aqueous solution, with zeta potentials of −24.5 ± 0.8 mV for HFn and 57.0 ± 1.7 mV for TAPC, respectively (Figure 2c). On this basis, we first mixed HFn with excess TAPC. According to the statistical particle size distributions derived from the height signal map of AFM (Figure 2b), there was a significant increase in the size of the assembly system (51.7 ± 7.3 nm) compared with that of HFn (11.4 ± 1.7 nm) and TAPC micelles (6.3 ± 0.9 nm), confirming the formation of coassembly, which we named T@H. The hydrodynamic size detected by dynamic light scattering (DLS) further confirmed this result (Figure 2d). In addition, the zeta potential of T@H was 51.8 ± 2.6 mV, similar to that of the TAPC micelles (Figure 2c). This suggests that TAPC micelles are distributed on the outer surface of the T@H assembly, whereas HFns are located in the core.

On this basis, we investigated the detailed mechanisms of HFn‐TAPC assembly. The analysis of bio‐layer interferometry (BLI) demonstrated a strong affinity between TAPC and HFn (affinity constant *K*_D_ = 7.46 × 10^−7^ m), which provides a foundation for their spontaneous and stable coassembly (Table S1, Supporting Information). Moreover, MD simulations of HFn and TAPC micelles were further conducted to probe their molecular binding mode. Consistent with the experimental results, the equilibrated structures acquired by MD confirmed the shell‐core structure of the T@H coassembly with the surface display of TAPC micelles (Figure 2e; Figure S3, Supporting Information). Moreover, it was revealed that a network of electrostatic interactions and hydrogen bonds is fundamental for achieving stable coassembly of TAPC micelles and HFn nanocages. Specifically, the positively charged protonated amino groups in TAPC micelles and the negatively charged carboxyl groups of aspartic acid (Asp) and glutamic acid (Glu) residues in HFn facilitate spontaneous coassembly through electrostatic interactions. On the other hand, hydrogen bonds also play a key role. The nitrogen atom in the TAPC amino group (acting as a hydrogen bond acceptor) forms a hydrogen bond with the hydroxyl group of the carboxyl group in the Asp or Glu residue (as a donor). Additionally, the amino groups of TAPC can also act as hydrogen bond donors to interact with the carboxyl groups of Asp or Glu residues (as acceptors) or with the amide groups from asparagine (Asn) or glutamine (Gln) residues. Numerous hydrogen bonds between the amino groups in TAPC and the amino acid residues in HFn increase the stability of the assembly. Given all the results above, the coassembly mechanism of T@H can be summarized as follows: In an aqueous environment, the oppositely charged TAPC micelles and HFn nanocages spontaneously assemble through electrostatic interactions. Once coassembly occurs, a network of hydrogen bonds at the assembly interface is crucial for maintaining stability and integrity.

### 2.3 Investigating the HFn‐Based LBL Supramolecular Assembly of H@T@H

Considering the safety and tumor‐targeting requirements, the external display of HFn in the drug delivery system is more favorable. Thus, we investigated the feasibility of the external assembly of HFn on T@H. First, MD simulation revealed that the subsequently added HFn nanocage could attach stably to the outer surface of T@H and form two binding regions (Figure 2f; Figure S4, Supporting Information). At the binding interface, the outward‐facing amino groups of T@H that remain unoccupied by the core HFn could form hydrogen bonds with the carboxyl groups (from Asp and Glu) and amide groups (from Asn and Gln) of the additional HFn on the outer surface. This hydrogen bond network is capable of supporting the structural stability of the LBL supramolecular assembly.

On this basis, we developed a two‐step method to guide the HFn‐based LBL supramolecular assembly, as illustrated in Figure 2g. First, HFn was mixed with excess TAPC to obtain the T@H coassembly. With the structure of TAPC on the outside, the positively charged T@H was further mixed with HFn in the second step. The additional HFn can coat the surface of T@H, forming an ordered multilayer coassembly, which we named H@T@H. To screen the optimal preparation scheme of LBL assembly, we added varying amounts of HFn into the T@H system. As shown in Figure 2h, the particle size of the LBL assembly nanosystem gradually increased with the addition of HFn, indicating successful coassembly between HFn and T@H. Meanwhile, the addition of HFn gradually neutralized the surface positive potential of T@H, suggesting the external assembly of HFn. In this case, when the H@T@H system reaches electrical neutrality, the assembly driving force of electrostatic interactions disappears so that the subsequently added HFn cannot assemble on the surface. To confirm the saturation point of the LBL assembly, we determined the TAPC loading rate of the H@T@H assemblies. The concentrations of TAPC and HFn were quantified via high‐performance liquid chromatography (HPLC) and sodium dodecyl sulfate polyacrylamide gel electrophoresis (SDS‐PAGE), respectively. When the TAPC/HFn molar ratio was increased to 450:1, the surface potential of the H@T@H assembly reached electrically neutral, and the TAPC loading ratio was ≈300 TAPC molecules per HFn. After that, the addition of extra HFn could no longer induce significant changes in the loading ratio of TAPC/HFn (Figure S5, Supporting Information). Based on these results, the TAPC/HFn molar ratio of 450:1 was applied to prepare the LBL assembly of H@T@H.

The structural morphology of H@T@H was subsequently characterized. The transmission electron microscope (TEM) images revealed that H@T@H had a spherical nanostructure (Figure S6, Supporting Information). The surface potential of H@T@H was −0.4 ± 0.7 mV and the hydrodynamic particle size was 62.2 ± 1.0 nm in diameter, with a polydispersity index (PDI) of 0.15 (Figure 2i). The AFM height signal maps revealed a statistical particle size distribution for H@T@H of 60.8 ± 6.6 nm (Figure 2j). The increase in the particle size of H@T@H compared with that of T@H was less than the theoretical increase expected from the HFn shell, suggesting that the electrostatic‐based coassembly is a flexible aggregate. The external HFn shell is likely to mediate the redistribution of TAPC molecules on the negative interface of HFn, compressing the spatial volume of TAPC in the interlayer. In addition, we examined the secondary structure of HFn before and after coassembly with TAPC. Circular dichroism (CD) spectroscopy and quantitative analysis of the secondary structure revealed that the supramolecular assembly did not cause protein conformational change in HFn (Figure 2k; Figure S7, Supporting Information). This confirms that HFn‐TAPC binding depends on electrostatic interactions and is not involved in the formation of covalent bonds. This finding also indicated that TAPCs were not embedded into the protein structure of HFn to cause conformational changes. In the evaluation of storage stability in solution, the particle size and surface potential of H@T@H negligibly changed during the 7 days of storage at 4 ℃ or at room temperature (Figure 2l; Figure S8a, Supporting Information). Even after storage at 4 ℃ for 3 months, H@T@H maintained structural integrity without aggregating (Figure S8b, Supporting Information). Additionally, no obvious changes in the particle size or distribution uniformity of H@T@H were detected in the simulated serum environment (in a medium containing 10% FBS at 37 ℃), indicating good serum stability (Figure S9, Supporting Information). Taken together, these data demonstrated that the ferritin‐based two‐step LBL supramolecular assembly strategy can efficiently load and orderly assemble TAPC in the interlayer of coassembly to neutralize its surface positivity, forming the H@T@H assembly with satisfactory stability in different environments.

### 2.4 Investigating the Endocytosis Behavior of H@T@H

**Figure 3:**
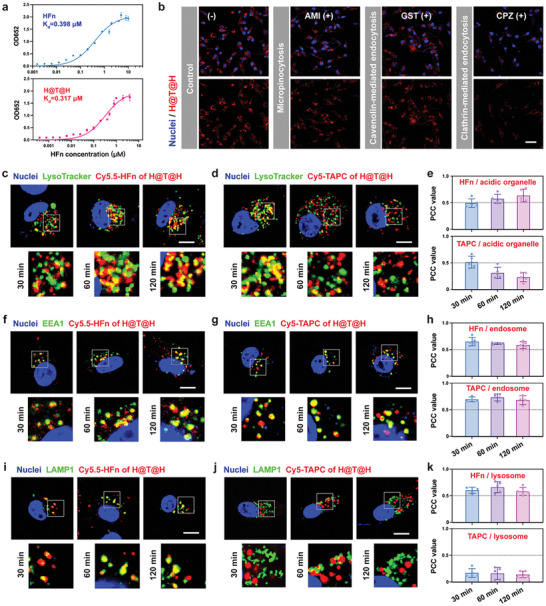
Investigating the endocytosis behavior and intracellular acid‐responsive disassembly of H@T@H. a) Analysis of the TfR1 affinity of HFn and H@T@H (*n* = 3). K_d_ (the equilibrium dissociation constant: ligand concentration that binds to half the receptor sites at equilibrium) was calculated by GraphPad. b) CLSM images of the internalized H@T@H in U87MG cells pretreated with different endocytosis inhibitors, scale bar = 50 µm. c–e) Colocalization analysis of Cy5.5‐HFn (c) or Cy5‐TAPC (d) of H@T@H with acidic organelles (indicated by LysoTracker) via CLSM and the corresponding quantitative analysis of PCCs (e), *n* = 5. f–h) Colocalization analysis of Cy5.5‐HFn (f) or Cy5‐TAPC (g) of H@T@H with early endosomes (indicated by EEA1) via CLSM and the corresponding quantitative analysis of PCCs (h), *n* = 4–5. i–k) Colocalization analysis of Cy5.5‐HFn (i) or Cy5‐TAPC (j) of H@T@H with lysosomes (indicated by LAMP1) via CLSM and the corresponding quantitative analysis of PCCs (k), *n* = 5. Scale bar = 10 µm. All data are presented as mean ± SD.

HFn has an inherent affinity for TfR1, which is essential for its receptor‐mediated endocytosis.^[^ ^28^, ^29^ ^]^ Therefore, we first assessed the TfR1 affinity of H@T@H using an enzyme‐linked immunosorbent assay (ELISA). The results revealed that H@T@H (K_d_ = 0.317 µm) exhibited TfR1 affinity comparable to that of wild‐type HFn (K_d_ = 0.398 µm) (**Figure** **3**a). Notably, the high TfR1 affinity of H@T@H was still maintained (K_d_ = 0.307 µm) after storage at 4 ℃ for 7 days, suggesting a stable coating of HFn on the T@H surface (Figure S10, Supporting Information). To study the uptake in tumor cells, Cy5‐labeled H@T@H was coincubated with human malignant glioblastoma cells (U87MG). As shown in the confocal laser scanning microscopy (CLSM) images, H@T@H could be rapidly internalized by tumor cells, reaching saturation at ≈2 h with a high intracellular TAPC level (Figure S11, Supporting Information). To confirm the endocytic pathway of H@T@H, we pretreated U87MG cells with different endocytosis inhibitors, including 1) amiloride (AMI), an inhibitor of pinocytosis; 2) genistein (GST), an inhibitor of caveolin‐mediated endocytosis; and 3) chlorpromazine (CPZ), an inhibitor of clathrin‐mediated endocytosis. Compared with the untreated group, the AMI and GST treatments had negligible effects on cellular uptake, whereas CPZ significantly reduced the intracellular accumulation of H@T@H (Figure 3b). These findings indicate that H@T@H was internalized by tumor cells through clathrin‐mediated endocytosis, which is consistent with the known TfR1‐mediated endocytic pathway of HFn.^[^ ^30^ ^]^ The above results demonstrated that H@T@H has the same TfR1 affinity and endocytosis pathway as HFn owing to the external assembly of HFn on the surface.

### 2.5 Acid‐Responsive Disassembly of H@T@H in Lysosomes

We subsequently prepared the fluorescence‐labeled H@T@H with Cy5.5‐HFn or Cy5‐TAPC to trace their intracellular behavior. First, the acid‐sensing fluorescent probe LysoTracker Green was employed to indicate the acidic organelles (including endosomes and lysosomes) in the cell. According to the CLSM images and the colocalization analysis of Pearson's correlation coefficient (PCC), the Cy5.5‐HFn of H@T@H was colocalized with LysoTracker at all times (Figure 3c,e; Figure S12a, Supporting Information). In contrast, the Cy5‐TAPC of H@T@H only colocalized with LysoTracker in the early stage (30 min). After that, the degree of colocalization gradually decreased (Figure 3d,e; Figure S12b, Supporting Information). These findings suggest that H@T@H undergoes disassembly during endosomal/lysosomal trafficking, leading to the divergent behaviors of TAPC and HFn in the cell. To further investigate the intracellular disassembly process, early endosome antigen 1 (EEA1) and lysosome‐associated membrane protein 1 (LAMP1) were used to identify early endosomes and lysosomes, which represent the early and late stages of endocytosis, respectively. First, we analyzed the endosome/lysosome localization of HFn in H@T@H. CLSM images revealed that Cy5.5‐HFn maintained a high degree of colocalization with both endosomes and lysosomes (Figure 3f,i; Figure S12c,e, Supporting Information). The PCC values were consistently >0.5 within 30–120 min (Figure 3h,k). In contrast, Cy5‐TAPC colocalized with only endosomes (PCC value > 0.5) (Figure 3g,h; Figure S12d, Supporting Information), which showed no significant colocalization with lysosomes (PCC value < 0.5) (Figure 3j,k; Figure S12f, Supporting Information). Based on these results, we speculated that H@T@H as a whole enters endosomes at the early stage of endocytosis. As endosomes mature into lysosomes with higher acidity, H@T@H undergoes acid‐responsive disassembly.

To verify this hypothesis, we prepared a bifluorescent‐labeled H@T@H to trace HFn and TAPC simultaneously. CLSM images at different time points (Figure S13, Supporting Information) revealed high colocalization of FITC‐HFn and Cy5‐TAPC in the initial stage of endocytosis (15–30 min). Subsequently, gradual separation of the two fluorescence signals was observed at 30–120 min, indicating the disassembly behavior of H@T@H in the acidic lysosomal environment. On this basis, we analyzed the TAPC release profile of H@T@H in different acidic pH solutions. The results demonstrated that the acidic environment promoted the disassembly of H@T@H, and the release rate and overall amount of TAPC increased with increasing acidity (Figure S14, Supporting Information). It can be explained by the isoelectric point (pI) of HFn being approximately pH 5–6. In lysosomes, the environmental pH is close to the pI of HFn. As a consequence, the surface negative charge of HFn diminishes, resulting in the weakening of electrostatic interactions between TAPC and HFn.

Taken together, the intracellular disassembly behavior of H@T@H can be summarized as follows: After endocytosis, TAPC and HFn initially enter early endosomes in the form of co‐assembly. Due to the relatively weakly acidic environment of endosomes (pH 6–6.5), significant disassembly of H@T@H does not occur. When TAPC enters the more acidic environment of lysosomes (pH 5–5.5), it rapidly weakens the electrostatic interaction between TAPC and HFn, accelerating disassembly and the release of TAPC from H@T@H. Finally, TAPC relies on its positive charges to achieve lysosomal escape.

### 2.6 In Vitro Evaluation of the Antitumor Activity of H@T@H

**Figure 4:**
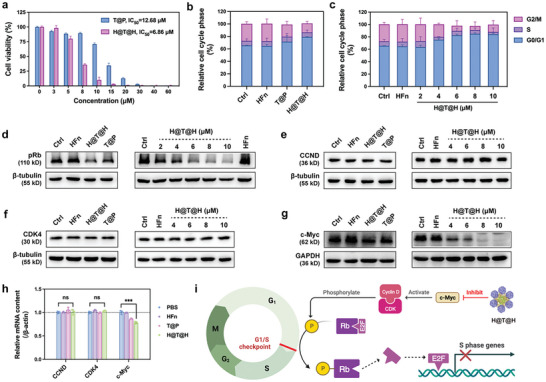
In vitro evaluation of the antitumor activity of H@T@H. a) Comparison of the cytotoxicity of T@P and H@T@H to tumor cells (*n* = 4). A CCK‐8 assay was used to determine the viability of U87MG cells treated with different TAPC formulations for 48 h. b,c) Cell cycle distribution of U87MG cells treated with different TAPC formulations (b) and different TAPC concentrations of H@T@H (c) for 30 h, *n* = 3. d–g) WB analysis of pRB (d), CCND (e), CDK4 (f) and c‐Myc (g) protein expression in U87MG cells treated with different TAPC formulations and different TAPC concentrations of H@T@H for 48 h. h) RT‐qPCR evaluation of the G1‐S transition associated mRNA content in the U87MG cells treated with different TAPC formulations for 24 h (*n* = 3). i) Schematic diagram of the H@T@H‐mediated cell cycle G0/G1 arrest. All data are shown as mean ± SD. *p*‐values were calculated via one‐way ANOVA with Tukey's multiple comparisons test, ^***^ *p* < 0.001, ns, not significant.

We subsequently evaluated the in vitro antitumor effects of H@T@H. Phosphorylated polyethylene glycol (PEG‐PO), which lacks antitumor activity and tumor‐targeting properties, was used to prepare PEG‐coated TAPC (T@P) as a control. The preparation and characterization of T@P are shown in Figure S15 (Supporting Information). First, we conducted a cytotoxicity evaluation on tumor cells. The results showed that H@T@H could effectively kill U87MG cells, with more potent cytotoxicity (IC_50_ = 6.86 µm) than T@P (IC_50_ = 12.68 µm) (**Figure** **4**a). In addition, H@T@H exhibited broad antitumor effects on different types of tumor cells (Figure S16, Supporting Information). Notably, the same concentration of H@T@H had no obvious toxic effect on normal cells, including human endothelial cells (HUVECs) and murine endothelial cells (bEnd.3). These findings suggested that H@T@H exhibits selective cytotoxicity on tumor cells, which can protect normal cells from undesired damage (Figure S17, Supporting Information).

Regarding the antitumor mechanism, flow cytometry analysis revealed that H@T@H triggered G0/G1 cell cycle arrest in a concentration‐dependent manner (Figure 4b,c). At the same concentration of TAPC, H@T@H resulted in 79.2% of the tumor cells arrested in the G1 phase, which was higher than that of T@P (71.7%). To investigate the underlying mechanism of G0/G1 arrest induced by H@T@H, we analyzed the expression levels of critical proteins involved in the G1 to S phase transition. Phosphorylated retinoblastoma (pRb) is a crucial regulatory protein that responsible for releasing the transcription factor E2F1/2/3 to initiate DNA replication and the G1/S transition.^[^ ^31^ ^]^ Western blot (WB) results demonstrated that H@T@H significantly downregulated the expression of pRb compared with T@P (Figure 4d; Figure S18a, Supporting Information). Next, we further analyzed the upstream regulators of pRb. During the G1/S transition in the G0/G1 phase, Cyclin D (CCND) forms a complex with cyclin‐dependent kinases (CDKs) in the nucleus, and the complex is essential for the phosphorylation of Rb.^[^ ^31^ ^]^ Results showed that H@T@H did not affect the protein expression of CCND or CDK4 in U87MG cells, even at high concentrations of TAPC (Figure 4e,f; Figure S18b,c, Supporting Information). Thus, we further studied c‐Myc, an essential early response protein for the G1/S transition. The results revealed that H@T@H treatment significantly inhibited the expression of c‐Myc in U87MG cells in a concentration‐dependent manner (Figure 4g; Figure S18d, Supporting Information). Moreover, RT‐qPCR was performed to analyze the mRNA levels of cell cycle‐related genes, including CCND, CDK4, and c‐Myc. Compared with the control group, H@T@H‐treated tumor cells (U87MG and HepG2) presented a significant decrease in the mRNA level of c‐Myc, with a more robust downregulation effect than did T@P‐treated cells (Figure 4h; Figure S19, Supporting Information). These results clarify the mechanism of H@T@H‐mediated G0/G1 arrest (Figure 4i). Specifically, H@T@H downregulates the expression of c‐Myc, impairing the activity of CCND/CDK4 complex and thereby impeding Rb phosphorylation. It prevents the release of transcription factors (e.g., E2F), ultimately hindering the cell cycle transition to S phase.

### 2.7 In Vitro Evaluation of the Anti‐Metastasis Effect of H@T@H

**Figure 5:**
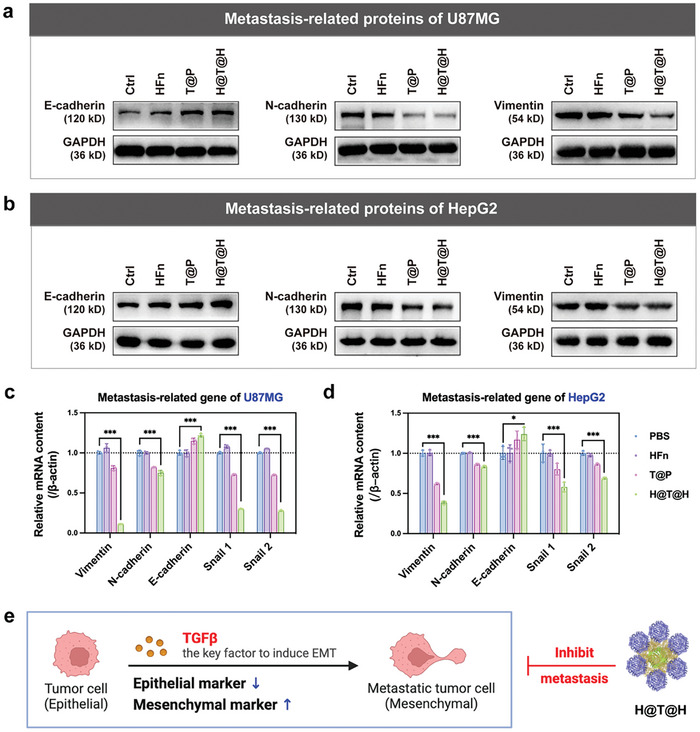
In vitro evaluation of the anti‐metastasis effect of H@T@H on various tumor cells. a,b) WB analysis of the expression of EMT‐associated proteins in U87MG (a) and HepG2 (b) cells treated with different TAPC formulations. c,d) RT‐qPCR analysis of the expression of metastasis‐associated mRNAs in U87MG (c) and HepG2 (d) cells treated with different TAPC formulations. Data are shown as mean ± SD (*n* = 3). *p*‐values were calculated via one‐way ANOVA with Tukey's multiple comparisons test, ^*^ *p* < 0.05, ^***^ *p* < 0.001. e) Schematic diagram of the antitumor metastasis effect of H@T@H.

The transforming growth factor β (TGFβ)‐mediated EMT of tumor cells plays an essential role in metastasis, which endows tumor cells with metastatic and invasive phenotypes, resulting in weakened cell adhesion, loss of polarity, and disruption of tight junctions.^[^ ^32^ ^]^ Therefore, we investigated the effect of H@T@H on the secretion of TGFβ by tumor cells. ELISA result demonstrated the decreased TGFβ levels in the culture supernatants of tumor cells treated with H@T@H (Figure S20, Supporting Information). Furthermore, we analyzed the EMT of tumor cells using WB. In both U87MG and HepG2 cells, H@T@H treatment of H@T@H significantly increased the expression of epithelial marker (E‐cadherin) and downregulated the expression of mesenchymal markers (N‐cadherin and vimentin) (**Figure** **5**a,b; Figure S21, Supporting Information). Similarly, RT‐qPCR was used to analyze the transcription levels of EMT‐related genes in tumor cells. Snail family members are essential transcription factors that up‐regulate in tumor cells undergoing EMT.^[^ ^33^ ^]^ Therefore, we also evaluated the mRNA levels of Snail 1 and Snail 2. Consistent with the WB results, tumor cells treated with H@T@H presented significantly increased transcription levels of epithelial‐related genes and decreased transcription levels of mesenchymal‐related genes. Additionally, the relative mRNA contents of Snail 1 and Snail 2 were significantly reduced. Notably, H@T@H treatment induced lower transcription levels of pro‐metastatic genes than did T@P treatment in various tumor cells (Figure 5c,d). These results demonstrated that H@T@H can reverse the EMT of tumor cells by inhibiting the release of TGFβ, thereby reducing the metastatic tendency of tumor cells (Figure 5e).

### 2.8 In Vivo Safety Evaluation of H@T@H

Prior to the in vivo evaluation of antitumor efficacy, the biosafety of H@T@H was evaluated. Healthy BALB/c mice were given a single injection of different dosages of H@T@H (2.5, 3.5, 5, or 10 µmol kg^−1^ TAPC) via the tail vein, with the saline administration group serving as a control. No weight loss was observed in the H@T@H‐treated group, with all the H@T@H‐treated groups exhibiting similar weight gain trends to those of the control group (Figure S22a, Supporting Information). On the 7th day, serum and blood samples were collected from the mice in each group. Serum biochemical analysis revealed that H@T@H‐treated mice had no abnormalities in the levels of biochemical indicators, including alanine aminotransferase (ALT), aspartate aminotransferase (AST), blood urea nitrogen (BUN), creatinine (CREA), and creatine kinase (CK) (Figure S22b–f, Supporting Information). In addition, blood routine tests revealed that the major blood cell counts in H@T@H‐treated mice were within the normal range (Figure S22g–l, Supporting Information). Notably, intravenous injection of high‐dose H@T@H (10 µmol kg^−1^ TAPC) had negligible toxic effects on the mice. In contrast, even a low dose of free TAPC (1 µmol kg^−1^) caused acute death in the mice. These results demonstrated that the HFn‐based supramolecular assembly drug delivery system can effectively enhance the biocompatibility of TAPC in vivo.

### 2.9 Specific Brain Tumor‐Targeting Ability of H@T@H In Vivo

**Figure 6:**
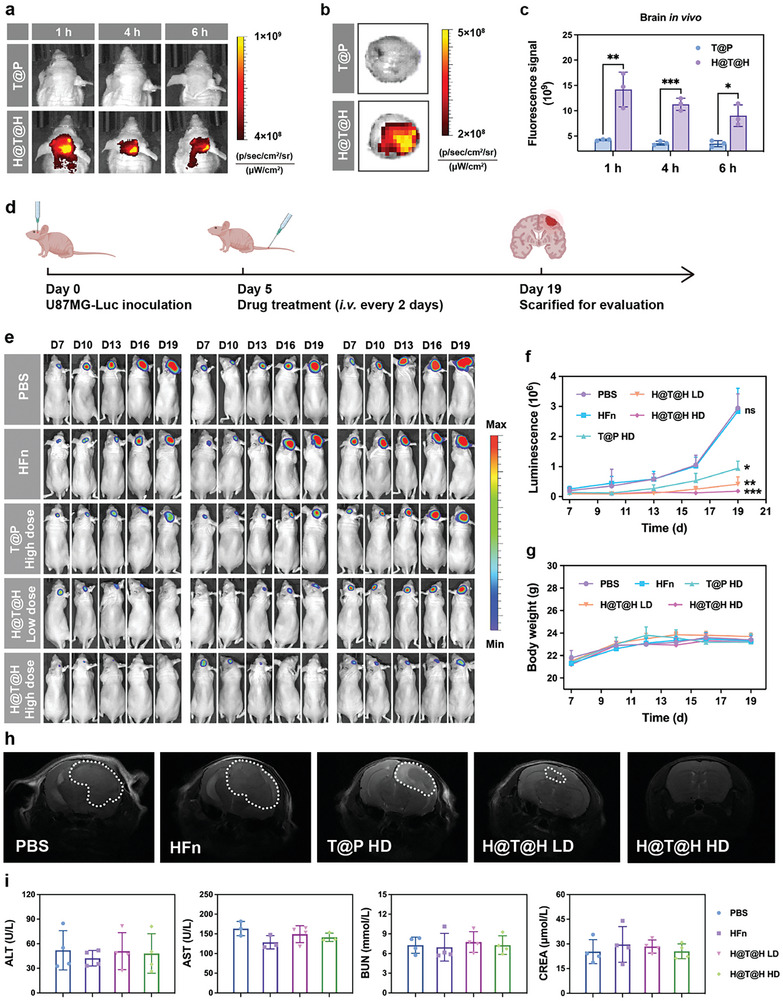
Evaluation of the in vivo distribution and antitumor efficacy of H@T@H in an orthotopic glioma model. a) Brain distribution of Cy5‐labeled T@P and H@T@H in orthotopic glioma‐bearing mice at different time points post‐administration. b) At 6 h after the administration of Cy5‐labeled T@P or H@T@H, the brain tissues of mice were dissected for ex vivo fluorescence imaging. c) Quantitative analysis of the accumulation of Cy5‐labeled T@P and H@T@H in the brains of mice. Data are shown as mean ± SD (*n* = 3). *P*‐values were calculated via unpaired two‐tailed Student's *t*‐test, ^*^ *p* < 0.05, ^**^ *p* < 0.01, ^***^ *p* < 0.001. d) Schematic diagram of the efficacy evaluation in the orthotopic glioma model. e) In vivo bioluminescence images of orthotopic glioma‐bearing mice in different treatment groups. f) Growth curves of U87MG‐Luc tumors in different treatment groups. Data are shown as mean ± SEM (*n* = 6). *p*‐values were calculated via one‐way ANOVA with Dunnett's multiple comparisons test by comparing the tumor luminescence of each group with that of the control (PBS group), ^*^ *p* < 0.05, ^**^ *p* < 0.01, ^***^ *p* < 0.001, ns: not significant. g) Body weight curves of mice in the different treatment groups. Data are shown as means ± SEM (*n* = 6). h) MRI of the brains in the different treated mice. i) Serum biochemical analysis of mice in different treatment groups. Data are shown as means ± SD (*n* = 4).

Studies have demonstrated that HFn can traverse the BBB and specifically target brain tumors.^[^ ^29^ ^]^ To verify the ability of H@T@H to cross the BBB, an in vitro transwell‐based BBB model was established (Figure S23a, Supporting Information). Human light chain ferritin (LFn), which has a similar structure to HFn but cannot traverse the BBB, was used as a negative control. The results demonstrated that H@T@H exhibited a significant transcytosis efficiency similar to that of HFn, whereas T@P failed to traverse BBB ECs (Figure S23b, Supporting Information). Next, the in vivo brain distribution was studied. Orthotopic glioma‐bearing mice were intravenously injected with Cy5‐labeled T@P or H@T@H, and then in vivo fluorescence imaging was conducted at 1, 4, and 6 h post‐injection. As shown in **Figure** **6**a, the TAPC fluorescence signal was almost undetectable in the brains of T@P‐treated mice. In contrast, by leveraging the inherent tumor‐targeting and BBB‐crossing abilities of HFn, H@T@H effectively accumulated in the brains of mice. Consistently, the ex vivo brain tissue of the H@T@H group exhibited stronger Cy5‐TAPC fluorescence than did that of the T@P group (Figure 6b). Quantitative analysis of fluorescence signals revealed a significantly greater accumulation of Cy5‐TAPC in the brains of H@T@H‐treated mice than in those of T@P‐treated mice (Figure 6c). In addition, matrix‐assisted laser desorption ionization time‐of‐flight mass spectrometry (MALDI‐TOF‐MS) was used to analyze homogenates of brain tumor tissue from the H@T@H‐treated mice. The TAPC molecule would be dissociated into C_60_ under the excitation of the high‐energy MALDI‐TOF‐MS laser. The characteristic MS signal of C_60_ (m/z:720) detected in the MS spectra further confirmed the effective brain delivery of TAPC mediated by the HFn‐based supramolecule assembly delivery system (Figure S24, Supporting Information).

### 2.10 In Vivo Antitumor Efficacy Evaluation of H@T@H in an Orthotopic Glioma Model

We subsequently evaluated the in vivo antitumor efficacy of H@T@H for the treatment of brain tumors. As illustrated in Figure 6d, an orthotopic glioma mouse model was established using a luciferase‐expressing glioma cell line (U87MG‐Luc). On the 5th day after glioma colonization, the mice were randomly divided into 5 groups. The mice in each group were intravenously injected every two days with PBS, HFn, high‐dose T@P (10 µmol kg^−1^ TAPC), low‐dose H@T@H (5 µmol kg^−1^ TAPC), or high‐dose H@T@H (10 µmol kg^−1^ TAPC). Glioma growth was monitored through in vivo bioluminescence imaging. The results demonstrated that HFn alone had no significant inhibitory effect on tumors, whereas T@P and H@T@H effectively suppressed glioma progression. The luminescence signal of the tumors in the H@T@H‐treated mice was lower than that in the T@P‐treated mice (Figure 6e). High‐dose H@T@H treatment resulted in nearly 93.8% inhibition of orthotopic glioma in mice. Notably, treatment with low‐dose H@T@H had a more significant inhibitory effect on brain tumors than did treatment with high‐dose T@P (Figure 6f). Magnetic resonance imaging (MRI) of brain performed on day 19 revealed that the glioma lesions in the H@T@H‐treated mice were much smaller than those in the other groups (Figure 6h). In terms of safety, the average body weight of H@T@H‐treated mice was comparable to that of the control group (Figure 6g). In addition, biochemical analysis of serum samples revealed the normal ranges of liver function indexes (ALT and AST) and kidney function indexes (BUN and CREA) in mice treated with different doses of H@T@H (Figure 6i). Furthermore, the main tissues of the H@T@H‐treated mice maintained normal physiological morphologies (Figure S25, Supporting Information). These results demonstrated that H@T@H can effectively inhibit the growth of orthotopic gliomas with good in vivo safety.

### 2.11 In Vivo Anti‐Metastasis Efficacy of H@T@H

**Figure 7:**
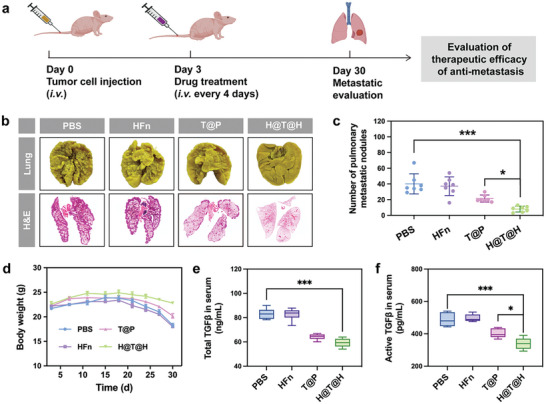
Evaluation of anti‐metastasis therapeutic efficacy. a) Schematic diagram of therapeutic efficacy evaluation in an experimental metastasis model of HepG2 cells. b) Representative photographs and panoramic scan images of H&E‐stained lung sections from mice subjected to different treatments. c) Number of lung metastatic nodules in each group. Data are shown as means ± SEM (*n* = 7). d) Body weight curves of the mice in each group. Data are shown as means ± SEM (*n* = 7). e–f) Secretion and activation of TGFβ in vivo. The serum concentrations of total TGFβ (e) and activated TGFβ (f) in each group were measured via ELISA. Data are shown as mean ± SD (*n* = 3). *p*‐values were calculated via one‐way ANOVA with Dunnett's multiple comparisons test, ^*^ *p* < 0.05, ^***^ *p* < 0.001.

Given the potential anti‐metastasis ability of H@T@H, as demonstrated in vitro by its ability to inhibit TGFβ secretion and reverse EMT of tumor cells, we further evaluated its in vivo anti‐metastasis efficacy in an experimental metastasis mouse model (**Figure** **7**a). On day 0, acute metastases were established by injecting HepG2 cells into the tail vein of BALB/c‐nu mice. Starting from day 3, the mice in each group were intravenously injected with PBS, HFn, T@P, or H@T@H. On day 30, the lung tissues of mice were collected to analyze the pulmonary metastatic nodules, and hematoxylin & eosin (H&E)‐stained sections of lungs were also prepared to observe internal metastasis. As shown in Figure 7b, there were numerous metastatic nodules in the lungs of the mice treated with PBS or HFn. In contrast, the H@T@H effectively inhibited pulmonary metastasis, with fewer and smaller lung metastatic areas than the T@P, which can be attributed to the tumor‐targeting ability of HFn. The quantitative analysis of metastatic nodules revealed that the metastatic inhibition rate of H@T@H was >80% (Figure 7c). In addition, the body weights of mice in the PBS and HFn groups decreased rapidly in the later period owing to the progression of tumor metastasis, and the body weights of the T@P‐treated mice also tended to decrease. In contrast, the weights of the H@T@H‐treated mice remained relatively stable (Figure 7d). These results demonstrate that H@T@H can effectively inhibit the development of distant metastasis and improve the quality of life of mice. To confirm the effects of H@T@H on the secretion and activation of TGFβ in vivo, the serum level of TGFβ was further determined via ELISA. The results demonstrated that the total TGFβ and activated TGFβ levels in the serum of the H@T@H group were decreased, which were significantly lower than those in the T@P group (Figure 7e,f).

Given the seed‐soil hypothesis of tumor metastasis, the presence of TGFβ in the circulation can create a favorable metastatic environment for the distant colonization of circulating tumor cells.^[^ ^32^ ^]^ Encouraged by the inhibitory effect of H@T@H on TGFβ, we further explored whether H@T@H has a preventive effect on tumor metastasis. To evaluate preventive efficacy (Figure S26a, Supporting Information), BALB/c mice were pretreated with PBS, HFn, T@P, or H@T@H. The next day, CT26‐Luc cells were injected into the tail veins of mice to simulate the hematogenous metastasis of circulating tumor cells. Lung metastasis was then monitored and quantitated via in vivo luminescence imaging. Compared with the control, the TAPC treatment significantly reduced the distant colonization of CT26‐Luc cells in the lung, with H@T@H showing better anti‐metastatic efficacy than T@P (Figure S26b,d, Supporting Information). H&E‐stained sections of lung tissue further confirmed that H@T@H‐pretreated mice had the lowest level of lung metastasis (Figure S26e, Supporting Information). The change in body weight was consistent with the progression of metastasis in each group (Figure S26c, Supporting Information).

Taken together, these results indicated that H@T@H has both preventive and therapeutic effects on tumor anti‐metastasis, which can effectively prevent the distant colonization of circulating tumor cells and inhibit the growth of metastatic tumors.

### 2.12 Broad Application of the HFn‐Based Supramolecular Assembly Strategy for Different Aminated Fullerene Derivatives

**Figure 8:**
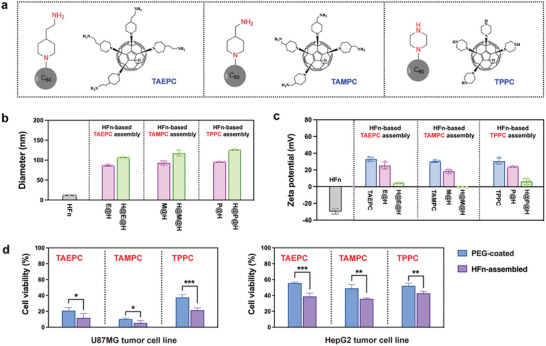
Investigation of the supramolecular assembly between HFn and different aminated fullerene derivatives. a) Molecular structures of different aminated fullerene derivatives, including TAEPC, TAMPC, and TPPC. b,c) Particle size (b) and zeta potential (c) of HFn‐based supramolecular assemblies with different aminated fullerenes (*n* = 3). d) Comparison of tumor cytotoxicity between the HFn‐assembled and PEG‐coated aminated fullerenes at the same concentration of TAPC. The viability of tumor cells was determined via a CCK‐8 assay after 24 h of different treatments. Data are shown as mean ± SD (*n* = 4). *p*‐values were calculated via unpaired two‐tailed Student's *t*‐test, ^*^ *p* < 0.05, ^**^ *p* < 0.01, ^***^ *p* < 0.001.

Furthermore, we investigated whether the HFn‐based supramolecular assembly strategy is applicable to other types of aminated fullerene derivatives. To this end, we additionally selected three aminated fullerene derivatives with different amination structures of side chains, including tetra[4‐(aminoethyl)piperidin‐1‐yl]‐C_60_ epoxide (TAEPC), tetra[4‐(aminomethyl)piperidin‐1‐yl]‐C_60_ epoxide (TAMPC), and tetra[4‐piperazin‐1‐yl]‐C_60_ epoxide (TPPC) (**Figure** **8**a; Figure S27, Supporting Information). TAEPC and TAMPC possess carbon chains of different lengths, which are aminoethyl and aminomethyl, respectively. Additionally, TPPC contains secondary amine groups, which are different from the primary amine groups of TAEPC and TAMPC. Zeta potential analysis revealed that all three derivatives had positively charged surfaces (Figure 8c).

The same LBL assembly method as TAPC was used to coassemble HFn with TAEPC, TAMPC, and TPPC. During the LBL assembly process, the changes in the particle size (Figure 8b) and surface potential (Figure 8c) of these aminated fullerenes were similar to those of TAPC, indicating the formation of “HFn‐aminated fullerene‐HFn” structures. The hydrodynamic diameter and PDI of these coassemblies were measured via DLS (Table S2, Supporting Information), which revealed uniform nanosizes (PDI < 0.2). CD analysis demonstrated that the protein conformation of HFn was not affected after coassembly with these aminated fullerenes (Figure S28, Supporting Information). The cytotoxicity evaluations of H@E@H, H@M@H, and H@P@H were conducted on U87MG and HepG2 tumor cells. Moreover, PEG‐PO was used to prepare the PEG‐coated preparations of TAEPC, TAMPC, and TPPC as controls (Figure S29, Supporting Information). As shown in Figure 8d, the HFn‐assembled aminated fullerene derivatives effectively killed tumor cells, resulting in a significantly stronger cytotoxic effect on tumor cells than the PEG‐coated derivatives did. These results confirmed the broad applicability of the HFn‐based electrostatic supramolecular assembly strategy for various aminated fullerene derivatives, indicating promising application prospects in tumor therapy.

## 3 Conclusion

This study aimed to address the in vivo application challenges of aminated fullerene derivatives associated with safety concerns derived from cation toxicity. We employed HFn as supramolecular assembly modules to construct a universal drug delivery system for aminated fullerene derivatives. The process and mechanism of HFn‐based LBL supramolecular assembly were elucidated through experimental characterization and MD simulations. Importantly, the ordered supramolecular assembly mode confers aminated fullerenes with reduced toxicity in vivo and enhanced tumor‐targeted therapeutic efficacy, facilitating the antitumor application of aminated fullerenes, especially for the treatment of brain tumors.

This study also provides new ideas for the development of ferritin drug carriers. The current drug‐loading strategy for ferritin is mainly disassembly/reassembly mediated internal encapsulation by virtue of its hollow cage‐like structure and reversible assembly property.^[^ ^26^ ^]^ In this study, we innovatively exploited the external drug‐loading potential of ferritin to develop a novel ferritin‐based drug delivery system by taking full advantage of the numerous potential molecular interactions on the external structure. Compared with traditional methods for drug loading of ferritin, the supramolecular assembly strategy is simple, fast, spontaneous, and controllable, without requiring complicated preparation steps or additional chemical reagents. This method also breaks the restriction of the limited inner space to achieve improvement in the amount and size of loaded drugs. Moreover, the electrostatic interaction‐mediated supramolecular assembly possesses flexibility and dynamicity that enable acid‐responsive disassembly and drug release, providing availability for precision medical treatment.

This ferritin‐based supramolecular assembly system also provides a potential platform for the combination chemotherapy of aminated fullerene derivatives. By comprehensively elucidating the antitumor mechanism of aminated fullerenes, drugs with synergistic effects can be further loaded into the cavity of ferritin to enhance the antitumor efficacy of externally assembled aminated fullerenes. Overall, this ferritin‐based supramolecular assembly provides a new paradigm for developing easy‐to‐prepare, broadly applicable, high‐loading, and targeted drug delivery systems.

## 4 Experimental Section

### Expression and Purification of the Recombinant HFn Protein

The amino acid sequence of HFn was obtained from NCBI (NCBI Reference Sequence: NP_002023.2). Recombinant HFn was expressed and purified as previously reported.^[^ ^29^ ^]^ The DNA sequence of HFn was subsequently cloned into the *Escherichia coli* (*E. coli*) expression vector pET30a plasmid (Novagen). The recombinant plasmid was transformed into *E. coli* competent BL21 cells (TransGen Biotech, Beijing) to express HFn protein. The transformed *E. coli* was cultured and proliferated in LB media supplemented with kanamycin. The expression of HFn protein was subsequently induced with 0.5 mm IPTG at 30 ℃ and 200 rpm for 8 h. The bacteria were collected via centrifugation and resuspended in 20 mm Tris‐HCl buffer (pH 8.0). Then, the resuspended bacterial mixture was passed through a high‐pressure homogenizer for bacterial lysis, and the upper layer of lysate was collected via centrifugation. For protein purification, most protein impurities were removed by heating at 72 ℃ for 15 min followed by centrifugation. The obtained supernatant was purified via anion exchange and size exclusion chromatography (GE Healthcare). Finally, the concentration of purified HFn was quantified with a BCA protein assay kit (Pierce).

### Preparation and Characterization of the HFn‐TAPC Supramolecular Assembly

6 mm TAPC was added to 2 µm HFn in an aqueous solution, and the mixed system was quickly vortexed. The obtained samples were subsequently centrifuged in ultrafiltration tubes (molecular weight cutoff 100 kDa) to remove unassembled TAPC molecules. After ultrafiltration, the sample was centrifuged at 12 000 rpm, and the obtained supernatant was T@H. A double volume of HFn (1 µm) was added to the T@H solution, and mixed thoroughly by vortexing. The obtained sample was centrifuged at 12 000 rpm, and the collected supernatant was H@T@H. The concentration of TAPC was determined via HPLC (Vanquish Core HPLC, Thermo Scientific). Concentration quantification of HFn was performed by grayscale of protein bands in SDS‐PAGE using the image processing software Image‐Pro Plus 6.0. For characterization, the morphology was characterized via a JEM‐1400 100 kV transmission electron microscope (JEOL, Japan). The particle size and surface potential were detected by a zeta potential and laser particle size analyzer (90Plus Zeta, Brookhaven). The protein conformation of HFn was examined by circular dichroism (Chirascan Plus) in the wavelength range of 190–260 nm, and the secondary structure was quantitatively analyzed via CNDD software.

### TfR1 Affinity Assay

The ELISA plate was precoated with human TfR1 protein (overnight at 4 ℃) and then blocked with PBS solution containing 0.5% BSA for 1 h at 37 ℃. Different concentrations of HFn and H@T@H were added to the wells and incubated at 37 ℃ for 1 h, followed by treatment with a mouse anti‐human HFn antibody (37 ℃ for 2 h) and HRP‐conjugated anti‐mouse IgG (37 ℃ for 2 h). Finally, the TMB substrate solution was added. After 30 min of incubation, the absorbance of each well at 652 nm was measured with a microplate reader (SpectraMax Plus384).

### Study of Intracellular Acid‐Responsive Disassembly

To trace the intracellular localization of TAPC and HFn in H@T@H, the Cy5‐TAPC, and Cy5.5‐HFn were used to prepare fluorescent‐labeled H@T@H, respectively. For the acidic organelle‐based study, the acid‐sensitive probe LysoTracker was adopted to indicate the acidic organelles in the cells. U87MG cells were cultured in confocal dishes and incubated with the fluorescent‐labeled H@T@H for 30, 60, or 120 min. In the last 30 min of coincubation, LysoTracker Green was added at a working concentration of 70 nm. Last, the cells were subsequently fixed with 4% paraformaldehyde, and the nuclei were stained with Hoechst 33342. The CLSM was used to analyze the colocalization of acidic organelles with Cy5‐TAPC or Cy5.5‐HFn.

For early endosome‐ and lysosome‐based studies, EEA1 and LAMP1 were adopted to indicate early endosomes and lysosomes in the cells. The drug treatment of cells was performed as described above. Then, cells were fixed with 4% paraformaldehyde, followed by permeabilization with 0.2% Triton X‐100. Next, cells were incubated with EEA1 (E9Q6G) mouse mAb (1:200 dilution, CST) or LAMP‐1 (H4A3) sc‐20011 (1:300 dilution, Santa Cruz) at 37 ℃ for 1 h and subsequently incubated with Alexa Fluor 488‐conjugated goat anti‐mouse IgG (H+L) (1:400 dilution, Life) for 1 h at 37 ℃. After the nuclei were stained with Hoechst 33 342, the colocalization was analyzed via CLSM.

### Cell Cycle Analysis

U87MG cells were cultured in 12‐well plates at a density of 2 × 10^5^ cells per well and then treated with PBS, HFn, 5 µm T@P, or 5 µm H@T@H. After 30 h of coincubation, cells in each group were treated with RNase at 37 ℃ for 30 min and stained with PI solution at 4 ℃ for 30 min. Finally, cells were resuspended in PBS buffer and detected by flow cytometry. The cell cycle analysis was performed with FlowJo software.

### Western Blot Analysis

Cells subjected to different treatments were lysed on ice with RIPA. Next, the cell lysate was mixed with 5× loading buffer and heated at 100 ℃ for 10 min. The protein samples were loaded and run on a 10% SDS‐PAGE gel and then transferred from gel to nitrocellulose membrane. The membrane was subsequently blocked with 5% skim milk for 1 h, incubated with primary antibodies at 4 ℃ overnight, and further incubated with HRP‐conjugated secondary antibody at room temperature for 1 h. Finally, the protein bands were visualized with an enhanced chemiluminescence western blot substrate (Tanon) and then imaged with an automatic chemiluminescence imaging analysis system (Tanon 4600). The WB results were quantified based on grayscale values of protein bands.

### Animals and Ethics Statement

BALB/c mice and BALB/c‐nu mice (male, 6–8 weeks old) were purchased from SPF Biotechnology Co., Ltd. (Beijing). All animal experiments were conducted according to the ethics protocol approved by the Institutional Animal Care and Use Committee at the Institute of Biophysics, Chinese Academy of Sciences (SYXK2021090).

### Brain Accumulation In Vivo

Mice bearing orthotopic U87MG gliomas were intravenously injected with equal TAPC doses of T@P and H@T@H, in which the TAPC was labeled with Cy5 fluorescence. At 1, 4, and 6 h post‐injection, the fluorescence signals of Cy5‐TAPC in the brains of mice were detected via an in vivo imaging system (IVIS, Lumina3). At 6 h after administration, the brain tissues were dissected and subjected to ex vivo imaging. At 12 h after administration, the brain homogenate was analyzed via MALDI‐TOF (ultrafleXtreme, Bruker).

### In Vivo Glioma Therapeutic Efficacy

The U87MG‐Luc orthotopic glioma model was established in healthy male BALB/c‐nu mice according to previous reports.^[^ ^29^ ^]^ To evaluate antitumor efficacy, the mice were randomly divided into 5 groups (*n* = 6): PBS, HFn, T@P HD, H@T@H LD, and H@T@H HD. The LD indicates a low dose of 5 µmol kg^−1^ TAPC equivalent, and the HD indicates a high dose of 10 µmol kg^−1^ TAPC equivalent. The mice in each group were intravenously injected with the corresponding drugs every two days. The bioluminescence signals of U87MG‐Luc tumors were detected via in vivo imaging every three days. On the 19th day, MRI analysis of the brains was performed. In addition, the H&E‐stained sections of brains were prepared to evaluate tumor progression, and the serum samples of mice were collected for serum biochemical analysis.

### Therapeutic Efficacy of Anti‐Metastasis

On day 0, the experimental metastatic model was established by intravenously injecting 5 × 10^5^ HepG2 cells into BALB/c‐nu mice. On day 3, the mice were randomly divided into 4 groups (*n* = 7) and intravenously administered PBS, HFn, T@P, or H@T@H every four days (5 µmol kg^−1^ TAPC equivalent). On day 30, the mice were sacrificed. The lung tissues of mice were collected and stained with Bouin's fixative solution to facilitate the observation of lung metastatic nodules. H&E‐stained lung sections were generated to evaluate metastasis within the tissues.

### Statistical Analysis

All data were analyzed via GraphPad Prism (GraphPad Inc., version 9). The statistical significance was analyzed by the unpaired two‐tailed Student's *t*‐test or one‐way analysis of variance (ANOVA). Data were shown as mean ± SD or mean ± SEM. In all cases, significance was defined as ^*^ *p* < 0.05, ^**^ *p* < 0.01, ^***^ *p* < 0.001, ^****^ *p* < 0.0001, ns, not significant.

## Conflict of Interest

The authors declare no conflict of interest.

## Author Contributions

B.Z. performed investigation, data curation, formal analysis, methodology, writing – original draft, wrote, reviewed, and edited the final manuscript. L.Y. performed investigation, formal analysis, wrote, reviewed, and edited the final manuscript. Y.J. performed investigation, methodology, formal analysis. Y.L., J.L., G.T., Y.L., and J.H. performed investigation. R.X. performed visualization. C.W. performed supervision, acquired funding acquisition, wrote, reviewed, and edited the final manuscript. X.Y. performed supervision, acquired funding acquisition, wrote, reviewed, and edited the final manuscript. J.L. performed conceptualization, supervision, data curation, acquired funding acquisition, wrote, reviewed, and edited the final manuscript. K.F. performed conceptualization, resources, supervision, data curation, acquired funding acquisition, project administration, wrote, reviewed, and edited the final manuscript.

## Supporting information



Supporting Information

## Data Availability

The data that support the findings of this study are available from the corresponding author upon reasonable request.
