# Supplementary material for: Ferritin‐Based Supramolecular Assembly Drug Delivery System for Aminated Fullerene Derivatives to Enhance Tumor‐Targeted Therapy
Source: Adv Sci (Weinh). 2024 Dec 31;12(8):2413389. doi: 10.1002/advs.202413389 (PMC11848615; doi:10.1002/advs.202413389)
Supplement: Supplementary file 1 — Supporting Information [file ADVS-12-2413389-s001.docx]

Supporting Information

Ferritin-based Supramolecular Assembly Drug Delivery System for Aminated Fullerene Derivatives to Enhance Tumor-targeted Therapy

Baoli Zhang, Libin Yang, Yiliang Jin, Yicheng Lu, Jianru Li, Guoheng Tang, Yang Liu, Jiawei Huo, Ran Xu, Chunru Wang*, Xiyun Yan*, Jie Li* and Kelong Fan*

B. Zhang

Zhejiang Provincial Key Laboratory of Pancreatic Disease

the First Affiliated Hospital, Zhejiang University School of Medicine

Hangzhou 310003, P. R. China

B. Zhang, Y. Jin, J. Li, G. Tang, R. Xu, X. Yan, K. Fan

CAS Engineering Laboratory for Nanozyme

Key Laboratory of Protein and Peptide Pharmaceutical Institute of Biophysics

Chinese Academy of Sciences

Beijing 100101, P. R. China

E-mail: yanxy@ibp.ac.cn; [fankelong@ibp.ac.cn](mailto:fankelong@ibp.ac.cn)

L. Yang, Y. Lu, Y. Liu, J. Huo, C. Wang, J. Li

Beijing National Research Center for Molecular Sciences

Key Laboratory of Molecular Nanostructure and Nanotechnology

Institute of Chemistry

Chinese Academy of Science

Beijing 100190, P. R. China

E-mail: crwang@iccas.ac.cn; [lijie24@iccas.ac.cn](mailto:lijie24@iccas.ac.cn)

L. Yang, Y. Jin, Y Lu, J. Li, G. Tang, R. Xu, C. Wang, X. Yan, K. Fan

University of Chinese Academy of Sciences

Chinese Academy of Sciences

Beijing 100408, P. R. China

X. Yan, K. Fan

Nanozyme Laboratory in Zhongyuan

Henan Academy of Innovations in Medical Science

Zhengzhou, Henan, 451163, P. R. China

**Supplementary Experimental Section**

**Materials**

Amiloride-HCl (AMI), genistein (GST), and chlorpromazine-HCl (CPZ) were purchased from Aladdin Scientific Corp. (Shanghai, China). The Cell Counting Kit-8 (CCK-8) and Cell Cycle Assay Kit-PI/RNase Staining were obtained from DOJINDO Chemical Technology (Shanghai) Co., Ltd. The mouse anti-EEA1 (E9Q6G) mAb, Cyclin D1 (E3P5S) XP® rabbit mAb, c-Myc (D84C12) rabbit mAb, and CDK4 (D9G3E) rabbit mAb were purchased from Cell Signaling Technology (CST, USA). E-cadherin polyclonal antibodies, N-cadherin polyclonal antibodies, and vimentin polyclonal antibodies were purchased from Proteintech (USA). LAMP-1 (H4A3) sc-20011 was purchased from Santa Cruz (USA). Beetle luciferin (potassium salt) was purchased from Promega (USA).

**Synthesis of aminated fullerene derivatives**

The aminated fullerene derivatives were synthesized using previously reported methods (Figure S1). ^[1]^ Take the synthesis of TAPC as an example. In detail, 80% CHP (3 eq.) and 4-(tert-butoxycarbonylamino)piperidine (15 eq.) were successively added to a C_60_Cl_6_ toluene solution, mixed and then stirred for 1 h at room temperature. The resulting solution was washed with 500 mL saturated NH_4_Cl and NaHCO_3_ aqueous solution. The organic phase was dried by anhydrous Na_2_SO_4_ and then concentrated with a rotary evaporator at 40℃. The crude product was eluted on a silica gel chromatography column with toluene-ethyl acetate mixtures to obtain purified TAPC-Boc. For deprotection, 100 mg TAPC-Boc was dissolved in 9 mL CH_2_Cl_2_, then 1 mL trifluoroacetic acid was added, and the mixture was stirred at room temperature for 2 h. The solvent was evaporated by a rotary evaporator at 30℃. An aqueous solution of the resulting trifluoroacetic acid salt was eluted on the ion exchange resin to replace CF_3_COO- with Cl-. Finally, the aqueous solution was filtered and freeze-dried to obtain an orange or brown powder of TAPC hydrochloride.

**Molecular dynamics simulations**

Owing to the large size of the HFn protein and micelle-like TAPC assembly, traditional molecular docking and MD simulation methods were unsuitable for approximating binding patterns between these molecules. Thus, we conducted progressive self-assembly molecular dynamics (PSMD) simulations via LAMMPS software to calculate and analyze the ideal binding modes.

First, for the self-assembly of amphiphilic TAPC, accurate TAPC-TAPC molecular docking was performed using AutoDock software. For the 1000 molecular docking system obtained, the cluster analysis module of AutoDock was applied to cluster the interaction phases, and the molecular docking energy was scored and ranked. The system with the maximum clustering phase and optimal energy scoring was selected for subsequent research. Based on the docking structure, the PSMD simulation based on the all-atom model (> 80 ns) was conducted via LAMMPS software. 79 TAPC molecules were randomly placed in a 10 nm × 10 nm TIP3P solvent box. PSMD simulation was also applied to calculate and analyze the LBL supramolecular assembly process as follows: (1) For the assembly of HFn and TAPC micelles, PSMD simulations (> 80 ns) were conducted via LAMMPS software. Twenty-five TAPC micelles were randomly placed in a TIP3P solvent box with the HFn protein at the center. (2) For the assembly of T@H and HFn, PSMD simulations (> 80 ns) were conducted via LAMMPS software. T@H and HFn were randomly placed in a TIP3P solvent box. In the PSMD simulations, the water box volume was continuously reduced every 10 ns, accelerating the self-assembly process.

The entire stimulation system was parameterized with a gaff force field, and the detailed PSMD simulation process was as follows: (1) Two-step energy minimization was performed. The molecule energy was minimized by initially constraining TAPC molecules (or TAPC micelles or T@H), followed by releasing them to minimize the energy of the entire system. The first energy minimization comprised 5000 cycles, with 1500 cycles utilizing the steepest descent method. The second energy minimization consisted of 5000 cycles, with 2000 cycles employing the steepest descent method. (2) NVT system equilibrium step was performed. During this step, the system underwent a heating equilibrium process maintained at 100 ps via the Langevin temperature control method. Subsequently, a 100 ps isotropic Berendsen pressure control method was applied for the pressure-equilibration process. (3) Unrestricted dynamic simulation stage. The temperature and pressure control methods are the same as those in the previous stage, with a cutoff distance of 10 Å for van der Waals energy and short-range electrostatic energy. The PME method is used to calculate the long-range electrostatic energy. In each step, the average structure was extracted at every 10 ns time step to analyze the conformation and binding modes between each TAPC molecule.

**Drug release behavior of H@T@H**

H@T@H was added to dialysis tubes (D-Tube Dialyzer Midi, MWCO 3.5 kDa) and then placed in buffer solutions of pH 5, pH 6, and pH 7.4. The dialysis tubes of each group were placed on a shaker (70 rpm) at room temperature. At different time points, the appropriate amount of dialysate was collected to quantify the TAPC release.

**Affinity assay of TAPC and HFn**

The affinity between TAPC and HFn was analyzed via biolayer interferometry (BLI). The His-tagged HFn (the N-terminus of HFn was fused with a His-rich sequence) was immobilized on the Ni-NTA sensor and sequentially bound to different concentrations of TAPC solution. The Octet® BLI molecular interaction analyzer Octet RED96e system was used to detect and analyze the binding and dissociation processes between HFn and TAPC.

**Cellular uptake and endocytosis pathway**

To evaluate cellular uptake, U87MG cells were cultured in confocal dishes and then incubated with H@Cy5-T@H at 37℃ for 15, 30, 60, 120, or 240 min. The cells were then fixed with 4% paraformaldehyde and stained with DAPI. Finally, the intracellular Cy5-TAPC fluorescence was observed via CLSM (Olympus, FV3000RS).

For analysis of the endocytosis pathway of H@T@H, U87MG cells were preincubated with different endocytosis inhibitors, including 100 μg mL^-1^ AMI, 50 μg mL^-1^ GST and 5 μg mL^-1^ CPZ, for 30 min. Then, the inhibitor-containing medium was replaced with the medium containing Cy5-H@T@H and incubated for 1.5 h. The following procedures for CLSM were the same as above.

**Intracellular disassembly of H@T@H**

The bifluorescence-labeled H@T@H was prepared by using Cy5-TAPC and FITC-HFn. U87MG cells were incubated with the bifluorescent H@T@H for 15, 30, 60, or 120 min. After coincubation, the cells were treated with fixation and nuclei staining. Finally, the CLSM was used to analyze the colocalization state of Cy5-TAPC and FITC-HFn.

**T@P preparation**

PEG-PO synthesis (Mw=1900) and the preparation of PEG-PO-coated TAPC were carried out as previously reported. ^[2]^ In detail, TAPC was added to an aqueous solution of PEG-PO (the molar ratio of PEG-PO to TAPC was 10:1) and vortexed for 10 s to obtain T@P.

**Cytotoxicity evaluation**

Tumor cells were cultured in 96-well plates at 8,000 cells per well and incubated with equal TAPC concentrations of T@P or H@T@H. After 24 h or 48 h of coincubation, the cell viability was evaluated with a CCK-8 assay kit according to the standard protocol.

**Real-time PCR analysis**

The tumor cells were treated with PBS, HFn, T@P, or H@T@H for 24 h. Total RNA was subsequently extracted from the cells with TRIzol reagent on ice. The extracted RNA was used as a template to synthesize DNA by strand-specific transcription based on an All-in-One 5× RT MasterMix kit (Abmgood, G490). Subsequently, qPCR reaction was performed using Taq Pro Universal SYBR qPCR Master Mix (Vazyme, Q172) to amplify the target DNA. β-actin was selected as the reference gene. The above experiments were carried out according to the manufacturer’s protocol. The sequences of primers are listed in Table S3.

***In vitro* BBB-crossing evaluation**

The *in vitro* BBB model was established as previously reported. ^[3]^ The mouse brain endothelial

cells (bEnd.3) were cultured on a Transwell plate with a 0.4 μm pore size (Corning, 3413) at a density of 6 × 10^4^ cells per well. When the transendothelial electrical resistance (TEER) of this BBB model reached 200 Ω·cm^2^, 2 μM Cy5.5-labeled LFn, Cy5.5-labeled HFn, Cy5-labeled H@T@H, or Cy5-labeled T@P was added to the upper chamber. After 2 h, the culture media in the lower chamber were collected, and their fluorescence was measured with an Enspire Multimode Plate Reader (PerkinElmer) to determine the transcytosis efficiency.

**Safety evaluation *in vivo***

Healthy BALB/c male mice were randomly divided into 5 groups (*n* = 4) and intravenously injected with 100 μL saline or different dosages of H@T@H (2.5, 3.5, 5, and 10 μmol kg^-1^, calculated as the dosage of TAPC). After that, the health status of the mice was monitored, and their body weights were recorded. One week later, whole blood samples were taken from the mice for blood routine tests to analyze the main blood cell levels. Moreover, serum samples were taken for serum biochemical analysis to evaluate the liver, renal, and myocardial functions.

**Preventive efficacy in anti-metastasis**

The male BALB/c mice were randomly divided into 4 groups (*n* = 5) and administered a single dose of PBS, HFn, T@P or H@T@H (5 μmol kg^-1^ TAPC equivalent). The next day, 5 × 10^5^ CT26-Luc cells were injected into the tail vein of the mice in each group. The bioluminescence signals of metastatic CT26-Luc cells in the lungs were detected using *in vivo* imaging of mice. On the 14th day, lung metastasis was evaluated using the method described above.

**Statistical analysis**

All data were analyzed via GraphPad Prism (GraphPad Inc., version 9). The statistical significance was analyzed by an unpaired two-tailed Student’s t-test or one-way analysis of variance (ANOVA). Data are shown as mean ± SD or mean ± SEM. In all cases, significance was defined as * *p* < 0.05, ** *p* < 0.01, *** *p* < 0.001, **** *p* < 0.0001, ns: not significant.

**Supplementary Figures**


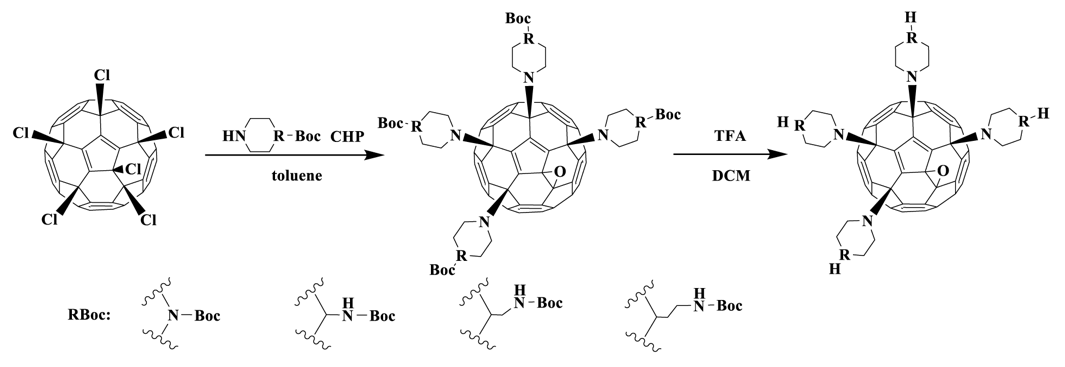


**Figure S1.** Synthetic route of aminated fullerene derivatives.


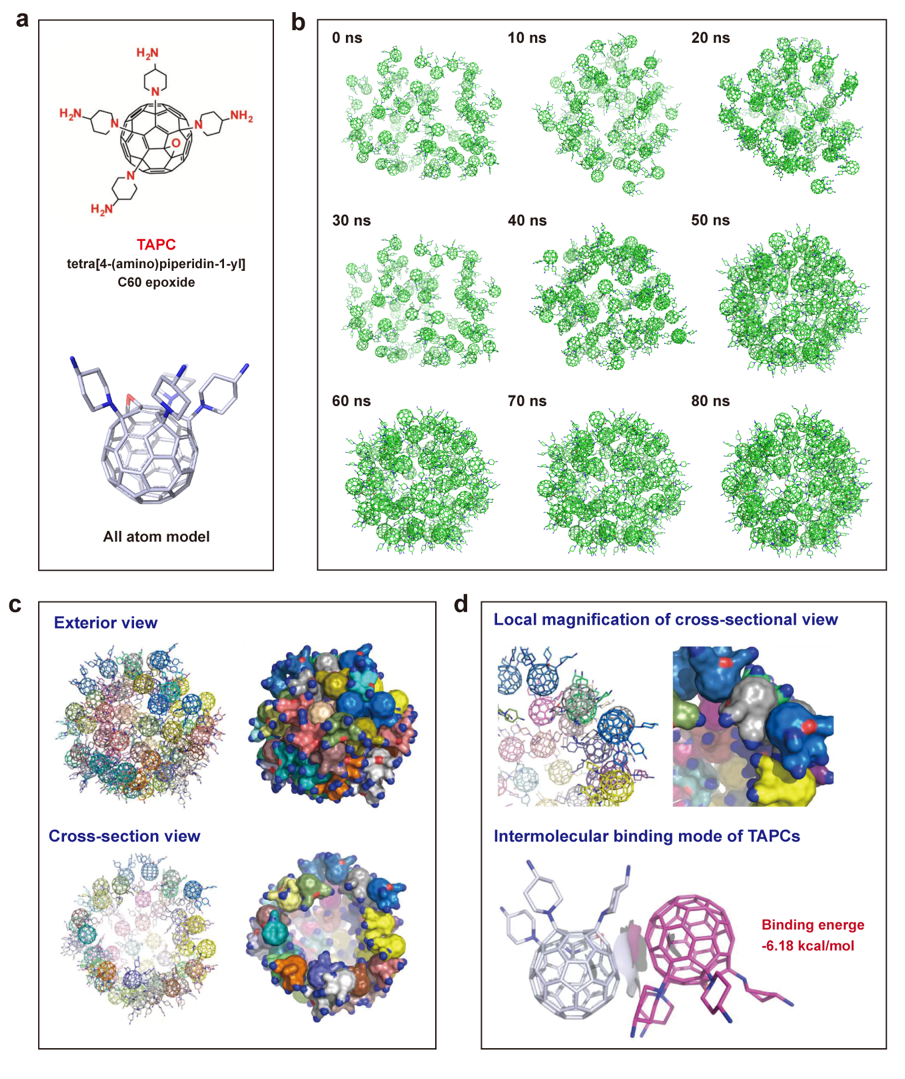


**Figure S2.** MD simulation of TAPC self-assembly. (a) Molecular structure of TAPC and its all-atom model for MD simulation. (b) During the MD simulation process, average structures of the system were acquired at an interval of 10 ns. Initially, from 0–10 ns, the TAPC molecules maintained a random distribution, remaining far from each other. Between 10–70 ns, the TAPC molecules began to approach each other, initially forming micelle-like structures. After 80 ns, the TAPC self-assembly reached a steady state, forming an ordered spherical micelle with a single-layer shell structure. (c) External and cross-sectional views of the TAPC assembly obtained from the MD simulation. Left: all-atom model. Right: surface model. (d) Local magnification of the cross-sectional view and the molecular binding modes between TAPC molecules.


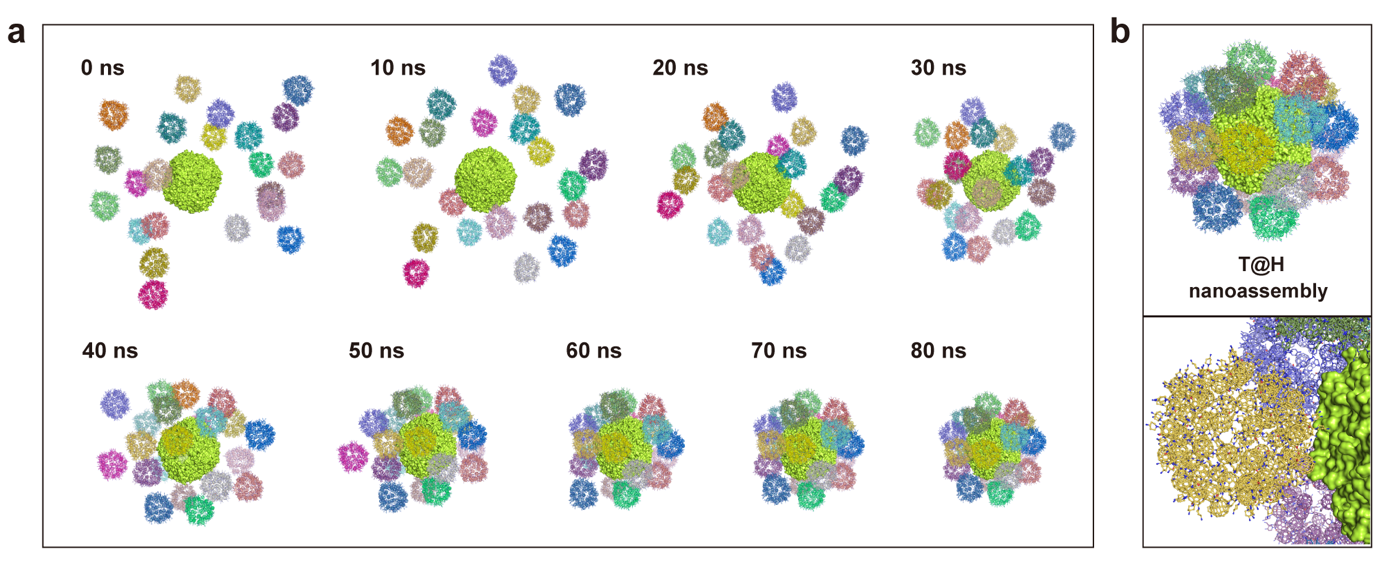


**Figure S3.** MD simulation of the supramolecular assembly of HFn and TAPC micelles. (a) Assembly states of TAPC-HFn at different time points during MD simulation. HFn is shown in green, and the individual TAPC micelles are distinguished by various colors to facilitate observation of their motion trajectory. At 0 ns, the TAPC micelles were randomly distributed in the solvent and far apart. Between 10–60 ns, the TAPC micelles gradually approached the HFn nanocage, forming a cluster-like structure. By 60–80 ns, the TAPC micelles moved closer to the central HFn, eventually forming a stable coassembly. (b) External view and local magnification view of the final stable structure of T@H.


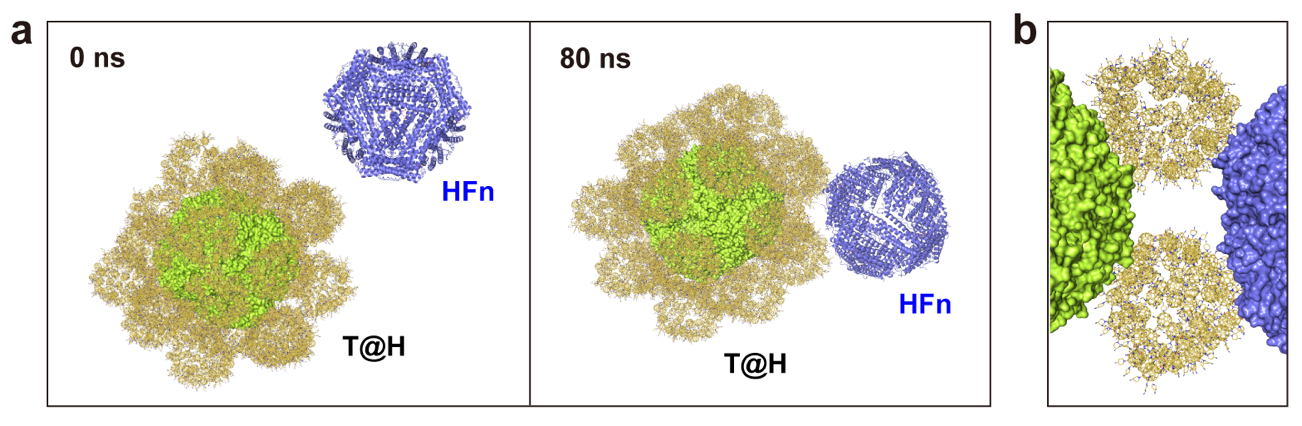


**Figure S4.** MD simulation of the coassembly of HFn and T@H. (a) Average structures of system acquired at 0 ns and 80 ns. (b) Magnified view of the assembly interface.


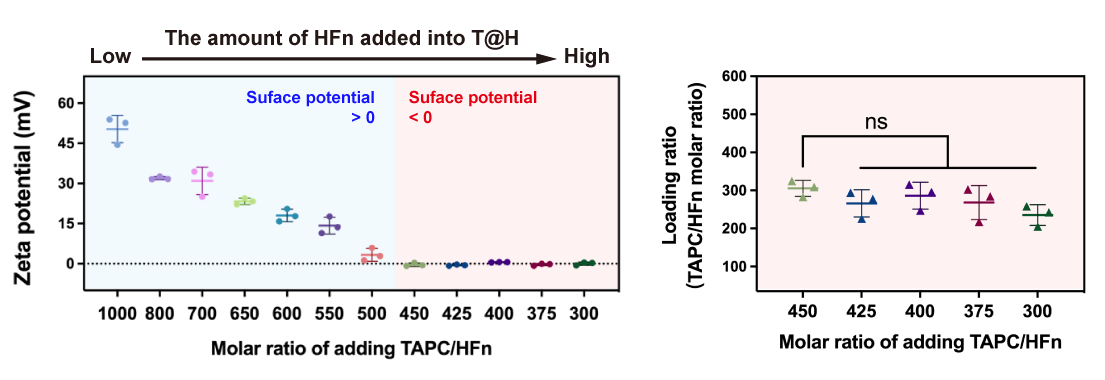


**Figure S5.** Investigation of the LBL assembly of HFn and T@H. Changes in surface potential during the LBL assembly of T@H with increasing addition amounts of HFn (Left) and the TAPC loading rates of assemblies with zeta potentials < 0 (Right). Data are shown as means ± SD (*n* = 3). *P*-values were calculated via one-way ANOVA with Tukey’s multiple comparisons test, ns: not significant.


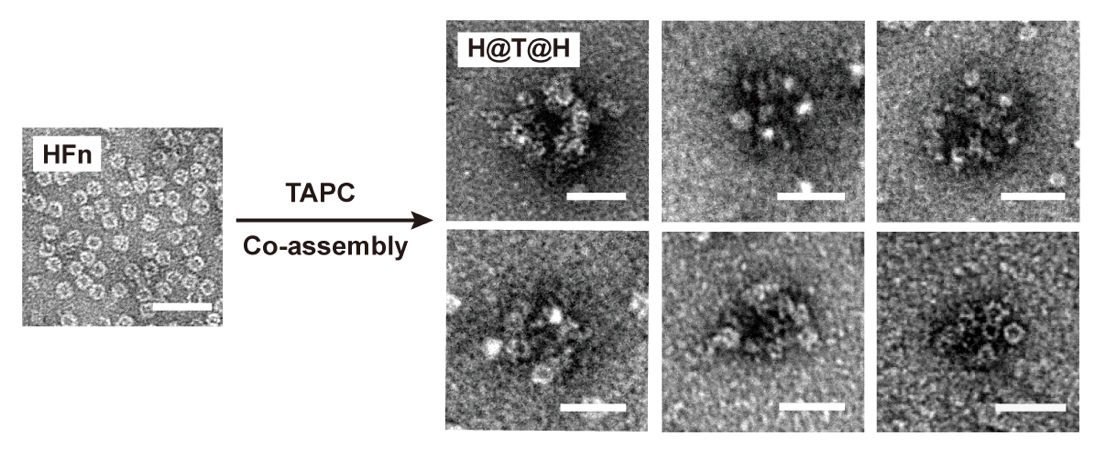


**Figure S6.** Morphology characterization by TEM. Representative TEM images of HFn and H@T@H, scale bar = 50 nm.


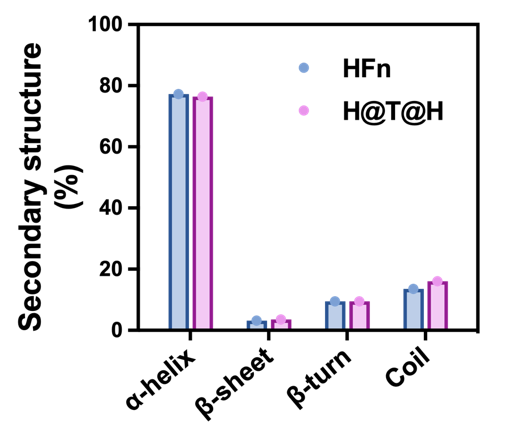


**Figure S7.** Quantitative secondary structure analysis of HFn and H@T@H according to CD spectra.


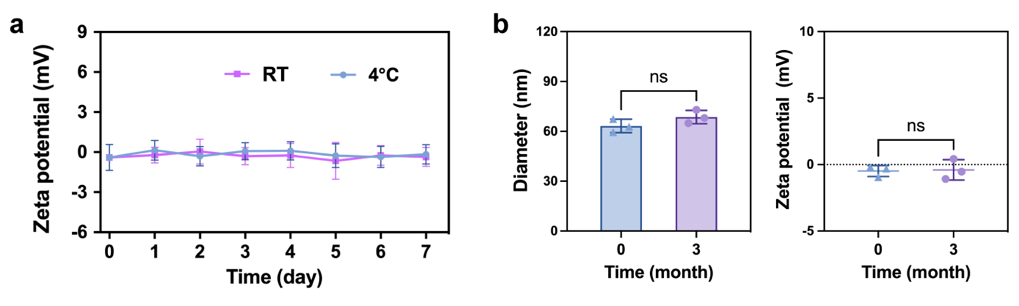


**Figure S8.** Stability evaluation of H@T@H in solution. (a) Zeta potential of H@T@H samples stored at 4℃ or room temperature for 7 days. (b) Particle size and surface potential of H@T@H before and after storage at 4℃ for 3 months. Data are shown as mean ± SD (*n* = 3). *P*-values were calculated via an unpaired two-tailed Student’s t-test, ns: not significant.


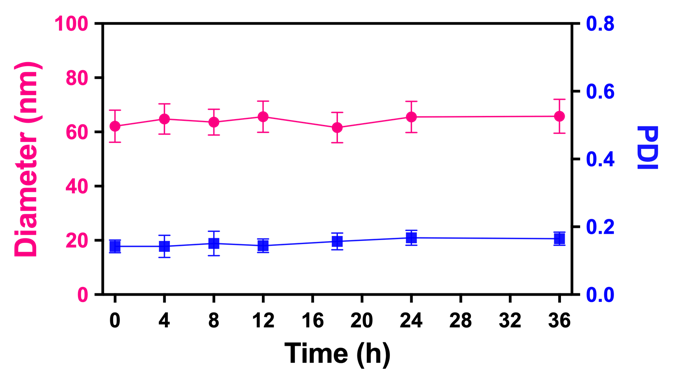


**Figure S9.** *In vitro* serum stability evaluation of H@T@H. H@T@H was placed in a medium supplemented with 10% FBS at 37℃, and its particle size and PDI were measured at different time points.


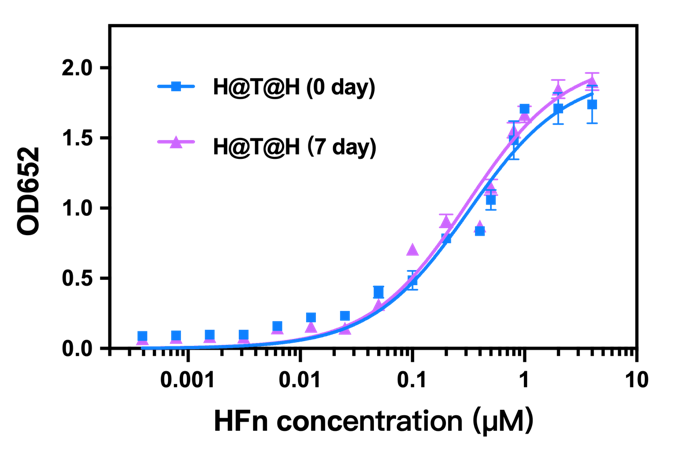


**Figure S10.** ELISA analysis of the TfR1 affinity of H@T@H stored at 4℃ for 0 and 7 days. Data are shown as means ± SD (*n* = 3).


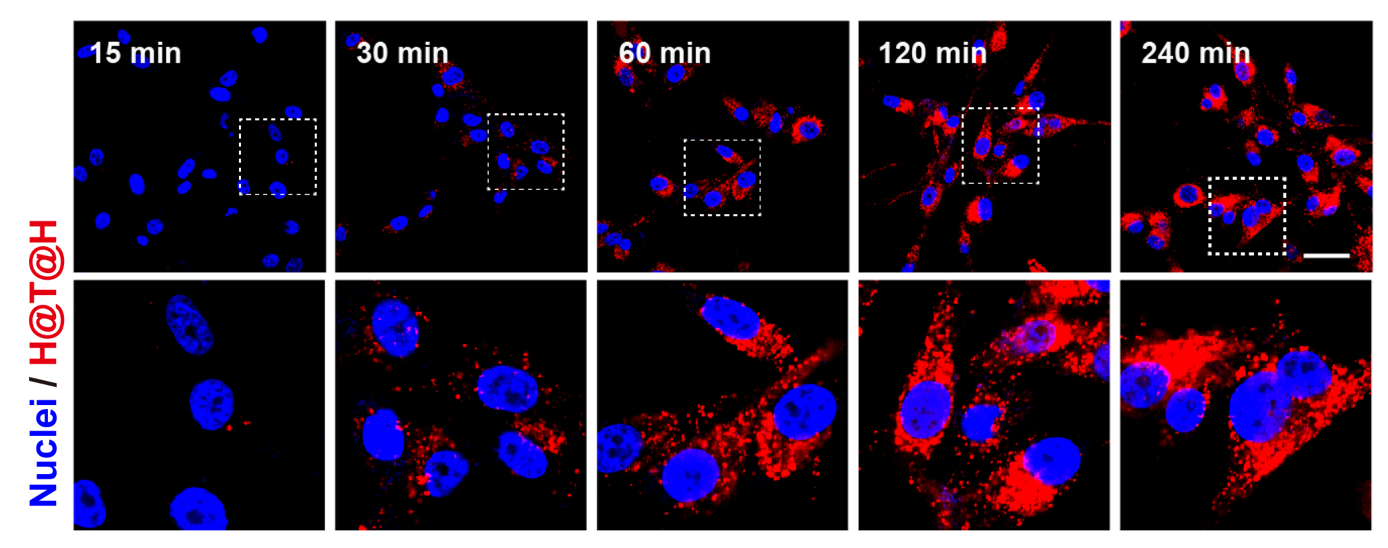


**Figure S11.** Evaluation of the cellular uptake of H@T@H. Cellular uptake of H@T@H in U87MG cells after coincubation for different times, scale bar = 40 μm.


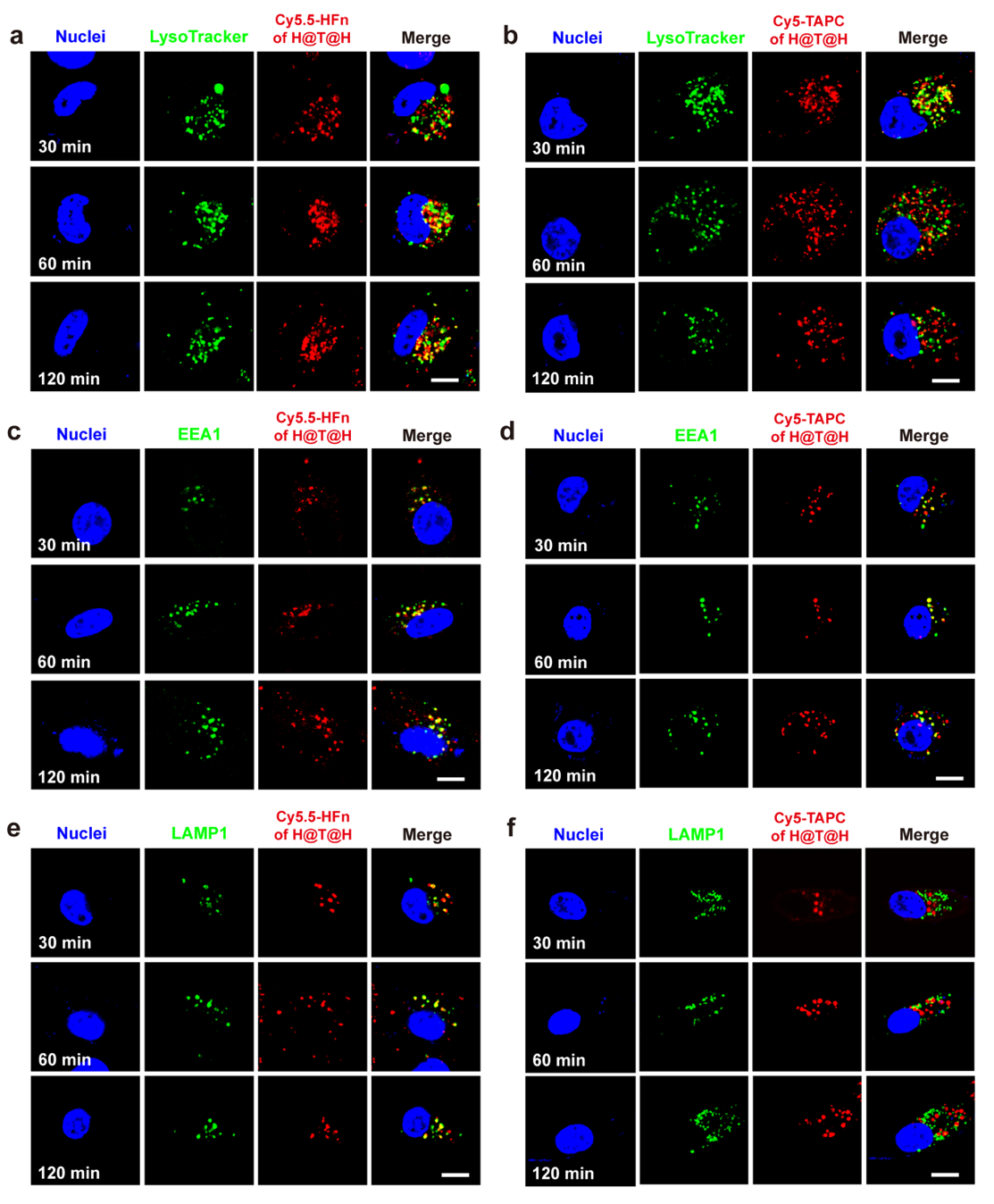


**Figure S12.** Intracellular localization of HFn or TAPC in H@T@H. Representative CLSM images of Cy5.5-HFn or Cy5-TAPC in H@T@H within U87MG cells after different incubation times. Figure S8a-f present single fluorescence channel images of Figure 3c, d, f, g, i, and j. Scale bar = 10 μm.


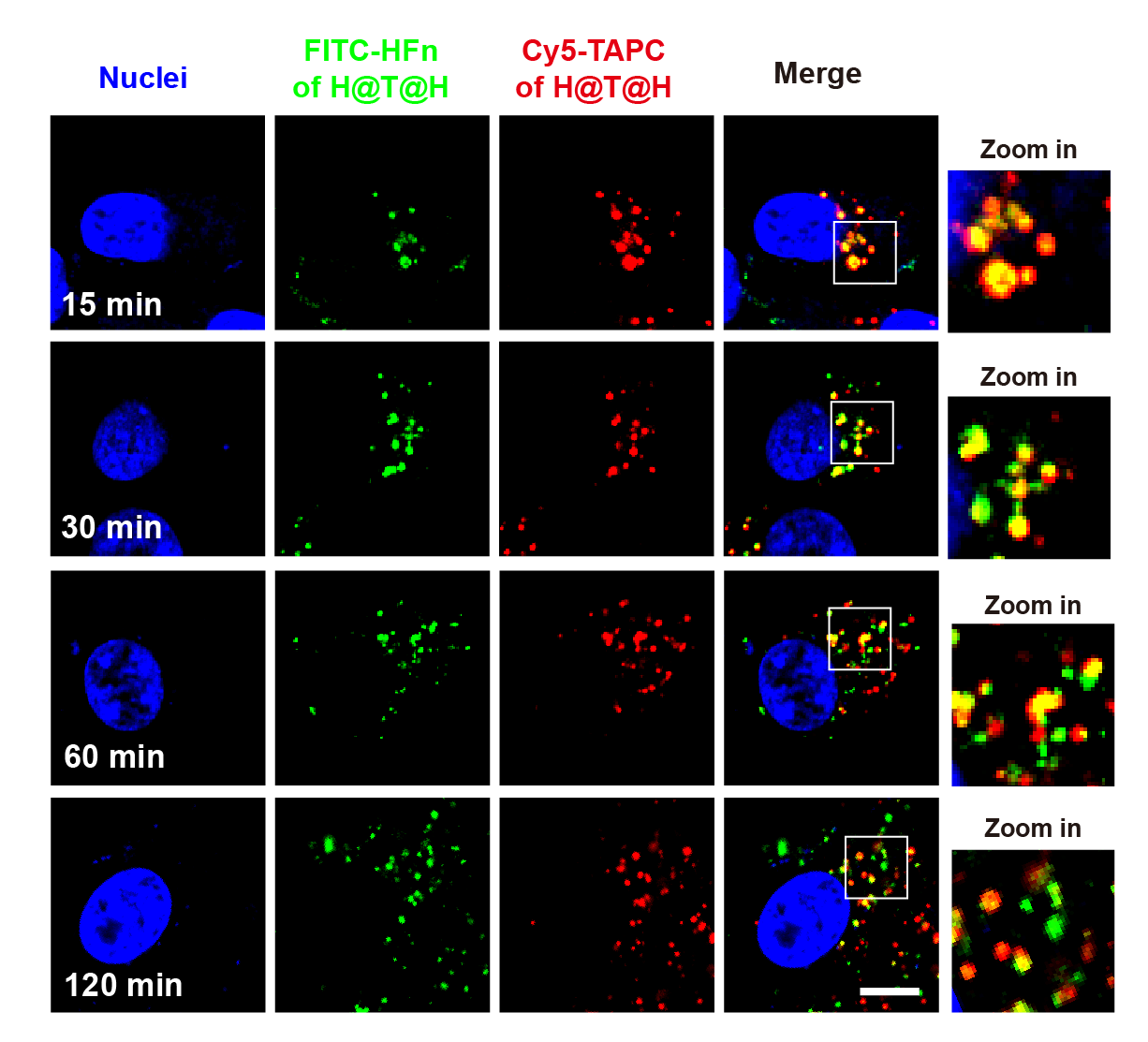


**Figure S13.** Intracellular disassembly of H@T@H. The bifluorescence H@T@H (prepared with FITC-HFn and Cy5-TAPC) was applied to investigate the intracellular disassembly process of TAPC and HFn in the U87MG cells. Scale bar = 10 μm.


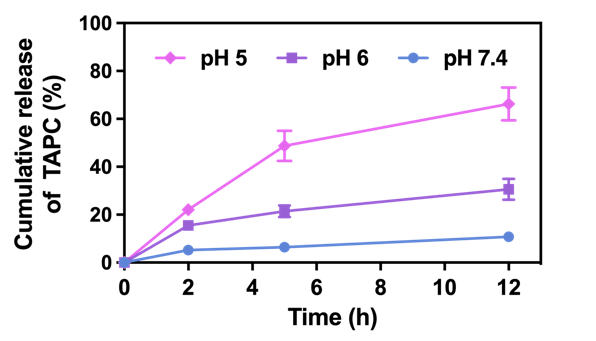


**Figure S14.** Evaluation of the acid-responsive disassembly behavior of H@T@H. TAPC release curves of H@T@H at pH 5, pH 6, and pH 7.4. Data are shown as means ± SD (*n* = 3).

**
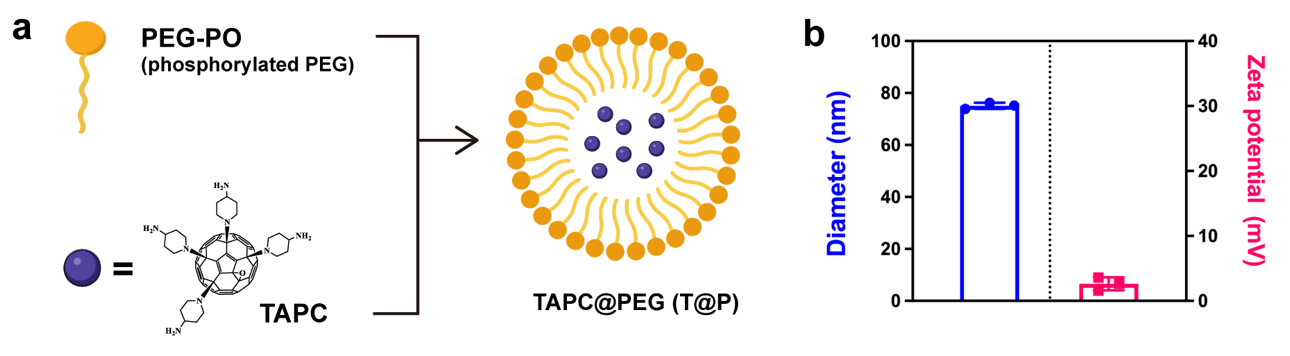
**

**Figure S15.** Preparation and characterization of T@P. (a) Schematic diagram of T@P preparation by coating TAPC with PEG-PO. (b) Characterization of the hydrodynamic diameter and zeta potential of T@P. Data are shown as means ± SD (*n* = 3).

**
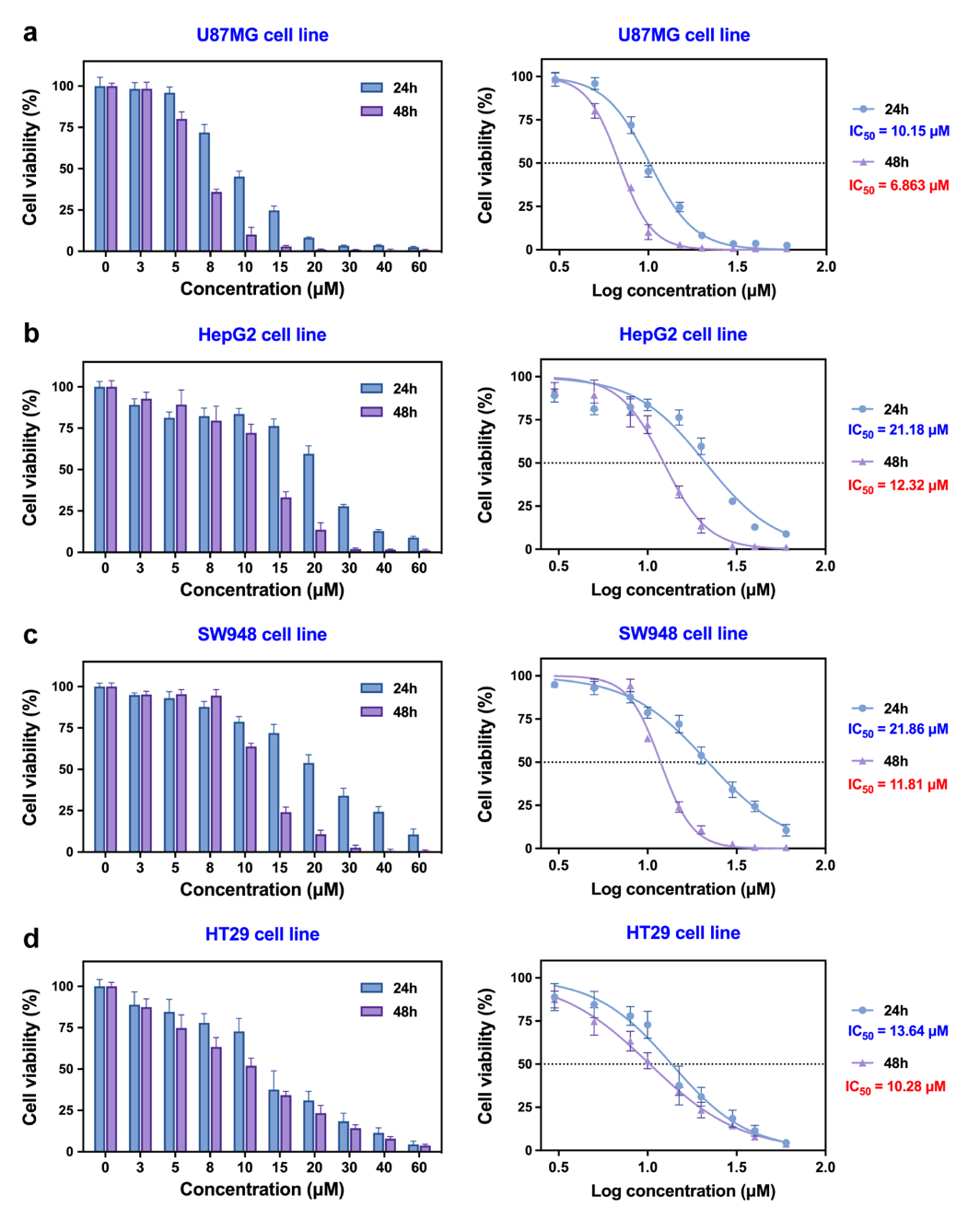
**

**Figure S16.** Evaluation of the antitumor effects of H@T@H on various tumor cell lines. (a-d) Cell viability of various tumor cells treated with H@T@H at various concentrations (determined by TAPC) for 24 h and 48 h, including U87MG (a), HepG2 (b), SW948 (c), and HT29 (d) cells, and their corresponding IC_50_ values were analyzed. Data are shown as means ± SD (*n* = 3).

**
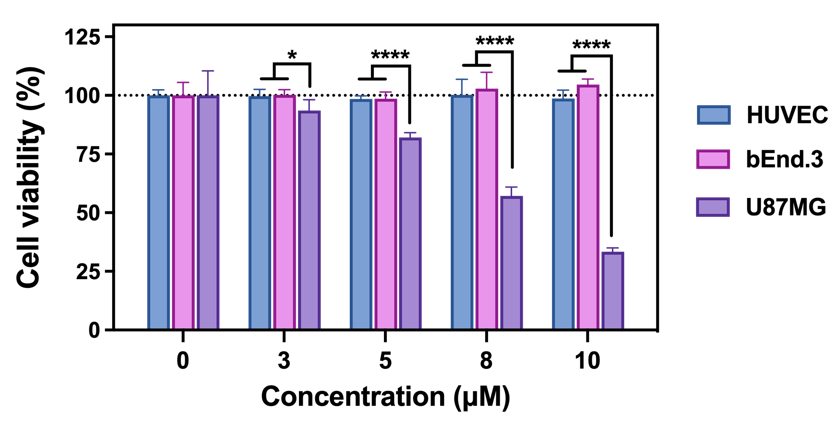
**

**Figure S17.** Comparison of the toxicity of H@T@H to tumor cells and normal cells. The viability of different cells treated with various concentrations of H@T@H (determined by TAPC) for 24 h, including human endothelial cell (HUVEC), mouse endothelial cell (bEnd.3), and human glioma cell (U87MG), was evaluated. Data are shown as means ± SD (*n* = 5). *P*-values were calculated via one-way ANOVA with Tukey’s multiple comparisons test, **p* < 0.05, *****p* < 0.0001.


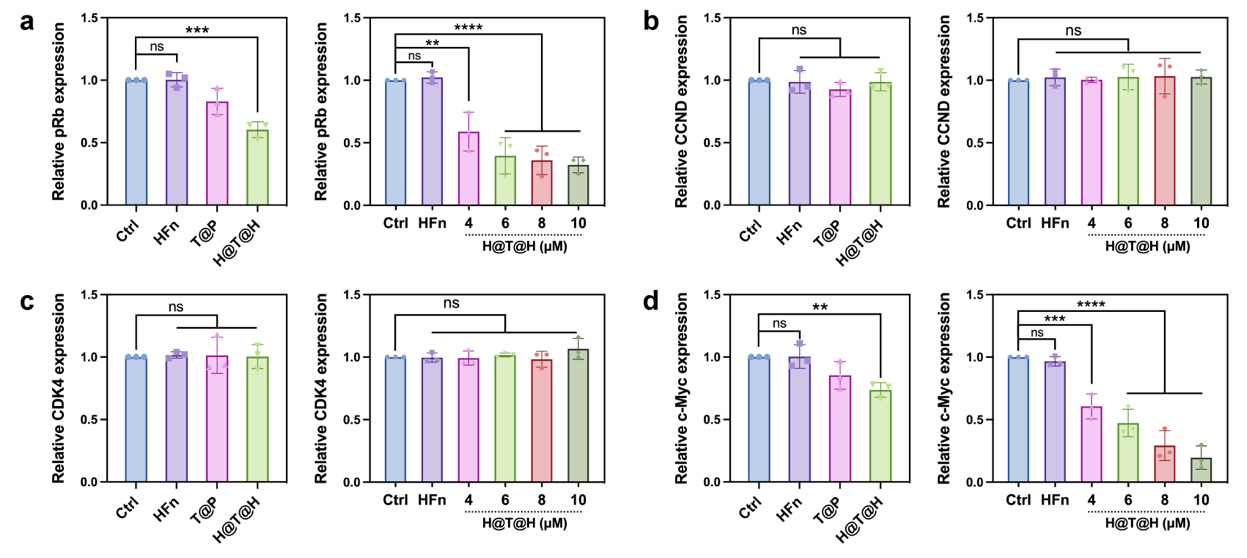


**Figure S18.** WB analysis of cell cycle-related protein expression in tumor cells. (a-d) Quantification of pRB (a), CCND (b), CDK4 (c) and c-Myc (d) protein expression in U87MG cells treated with different TAPC formulations or H@T@H at different concentrations (determined by TAPC). Data are shown as mean ± SD (*n* = 3). *P*-values were calculated via one-way ANOVA with Tukey’s multiple comparisons test, ***p* < 0.01, ****p* < 0.001, *****p* < 0.0001, ns: not significant.


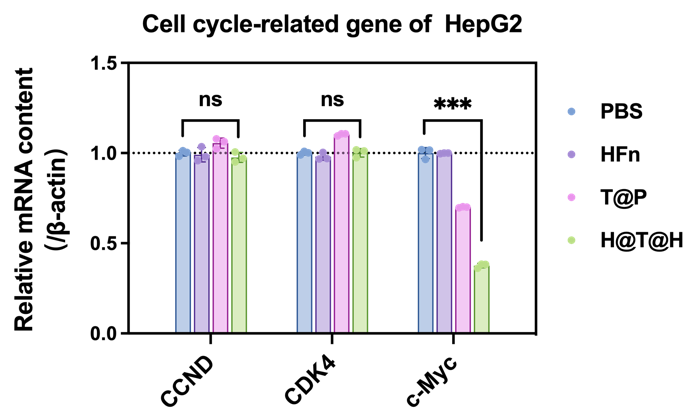


**Figure S19.** RT-qPCR evaluation of cell cycle-related genes in HepG2 cells. The HepG2 cells were treated with different TAPC formulations for 24 h, and the content of mRNAs associated with the G1 to S phase transition was analyzed via RT-qPCR. Data are shown as mean ± SD (*n* = 3). *P*-values were calculated via one-way ANOVA with Tukey’s multiple comparisons test, ****p* < 0.001, ns: not significant.


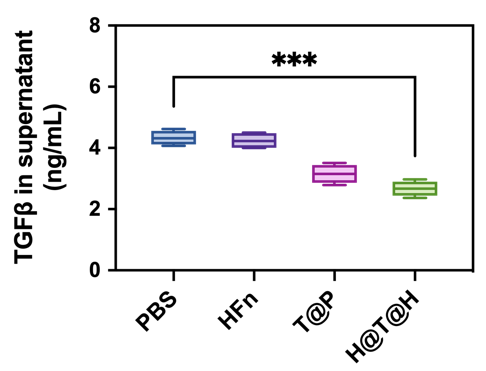


**Figure S20.** Effect of H@T@H on TGFβ secretion from tumor cells*.* The concentration of TGFβ in the culture supernatant of HepG2 cells was detected after different treatments for 48 h. Data are shown as mean ± SD (*n* = 4). *P*-values were calculated via one-way ANOVA with Tukey’s multiple comparisons test, ****p* < 0.001.


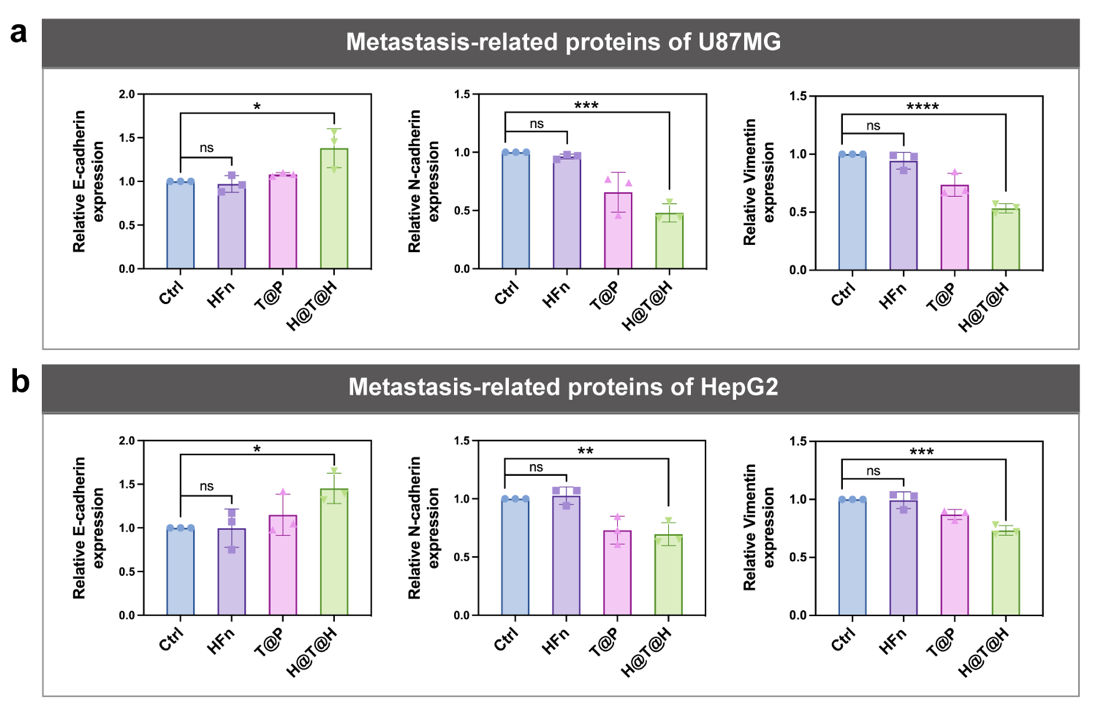


**Figure S21.** WB analysis of metastasis-related protein expression in tumor cells. (a-b) Quantification of metastasis-associated protein expression in U87MG (a) and HepG2 (b) cells treated with different TAPC formulations. Data are shown as mean ± SD (*n* = 3). *P*-values were calculated via one-way ANOVA with Tukey’s multiple comparisons test, **p* < 0.05, ***p* < 0.01, ****p* < 0.001, *****p* < 0.0001, ns: not significant.


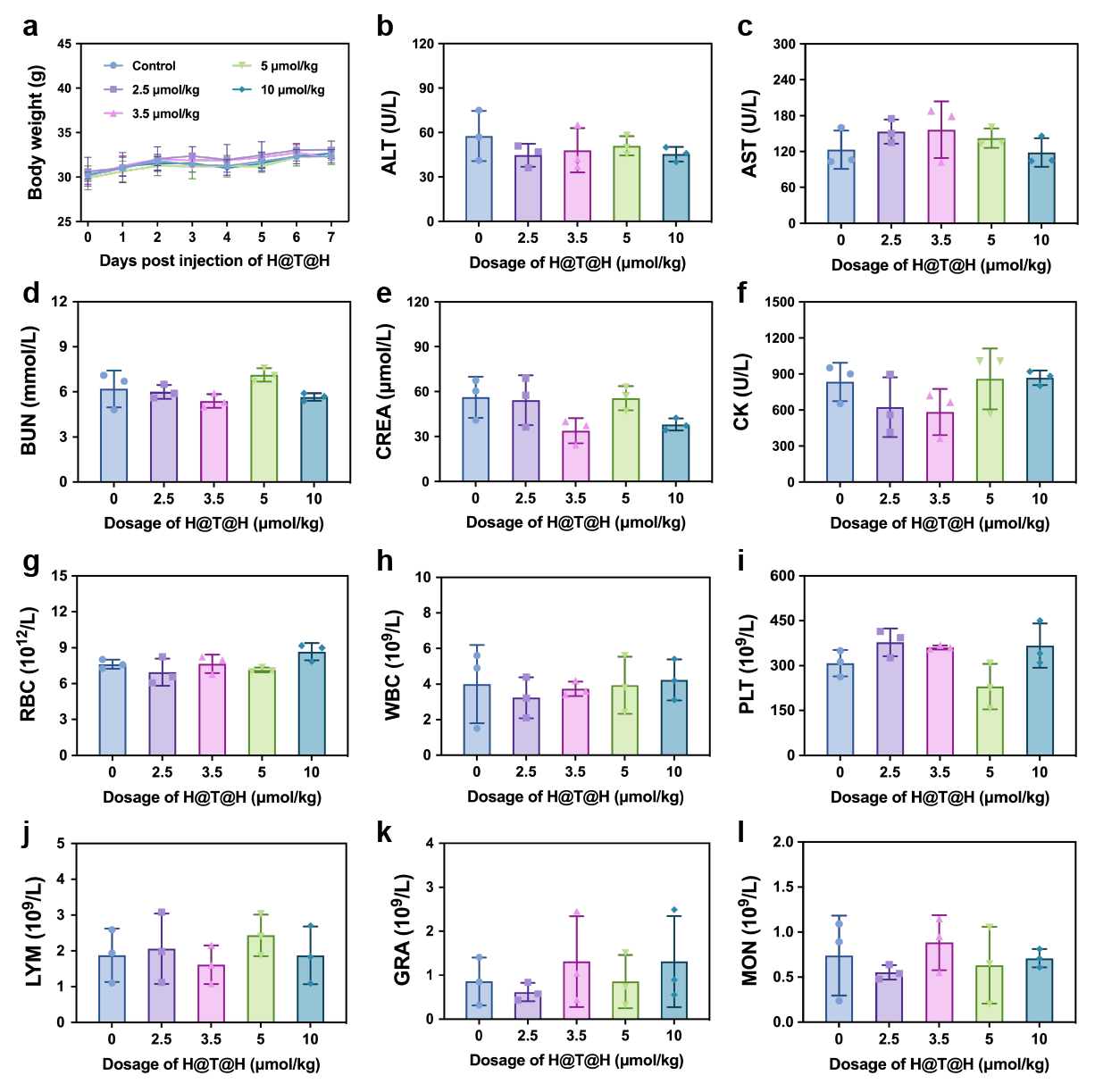


**Figure S22.** Biosafety study of H@T@H in healthy BALB/c mice. (a) Body weight curves of the mice after the administration of different dosages of H@T@H. (b-c) Serum biochemical analysis of liver function indicators. (d-e) Serum biochemical analysis of kidney function indicators. (f) Serum biochemical analysis of myocardial function indicators. (g-l) Blood routine assay of major blood cells in the blood, including red blood cells (g), white blood cells (h), platelets (i), lymphocytes (j), granulocytes (k), and monocytes (l). The dosages of H@T@H were determined by TAPC. All data are shown as means ± SD (*n* = 3).

**
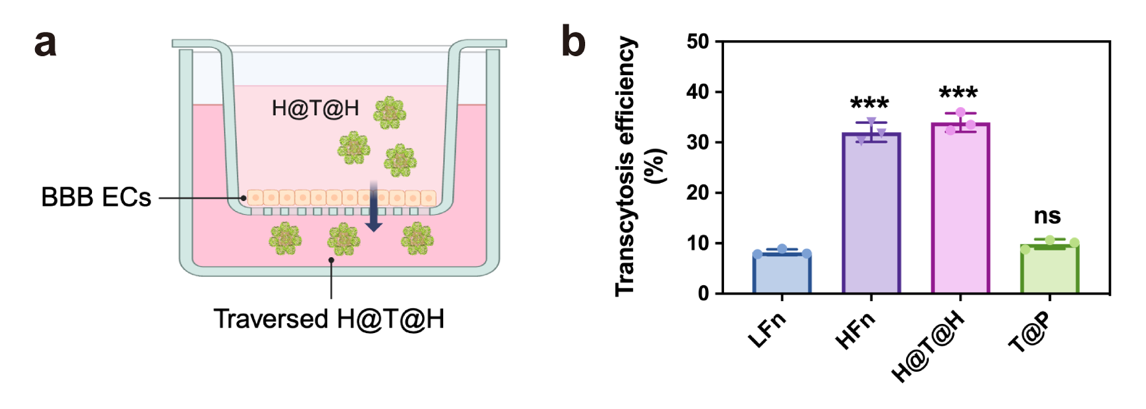
**

**Figure S23.** *In vitro* evaluation of the BBB-crossing ability of H@T@H. (a) Schematic illustration of the *in vitro* BBB model. (b) Transcytosis analysis of LFn, HFn, and different TAPC formulations. Data are shown as means ± SD (*n* = 3). *P*-values were calculated via one-way ANOVA with Tukey’s multiple comparisons test compared with the control (LFn), ****p* < 0.001, ns: not significant.


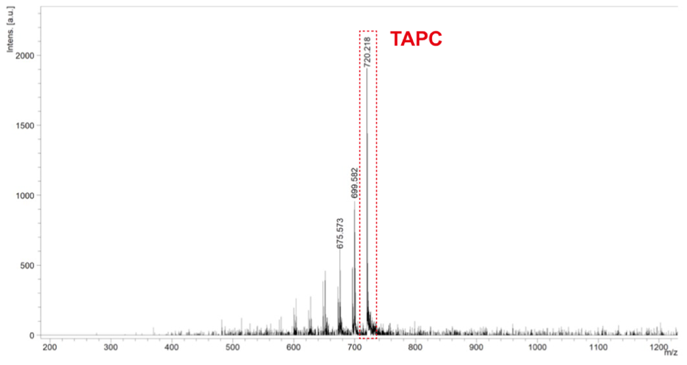


**Figure S24.** MALDI-TOF-MS detection of brain tissue homogenates of H@T@H-treated mice.


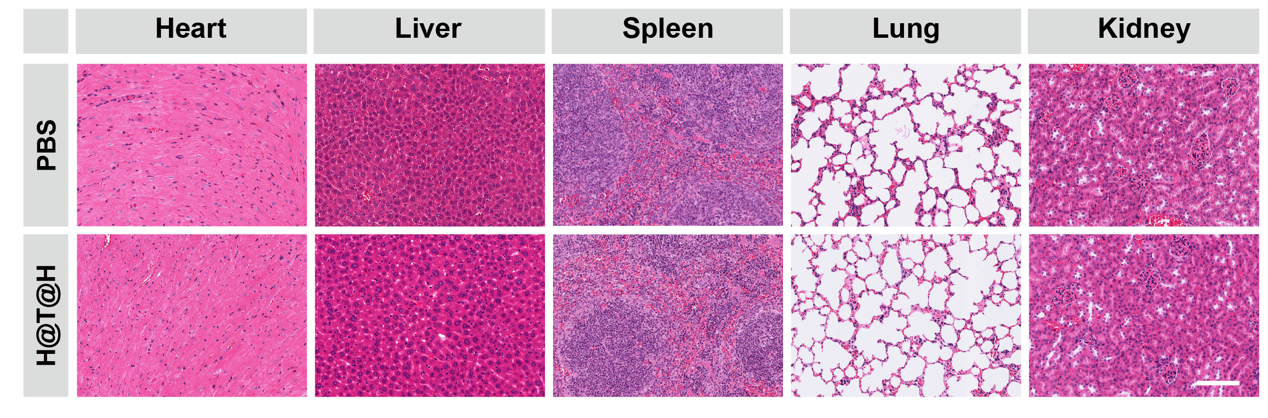


**Figure S25.** Safety evaluation of H@T@H in orthotopic glioma therapy. H&E-stained sections of main tissues from the mice treated with PBS or H@T@H, scale bar = 100 μm.


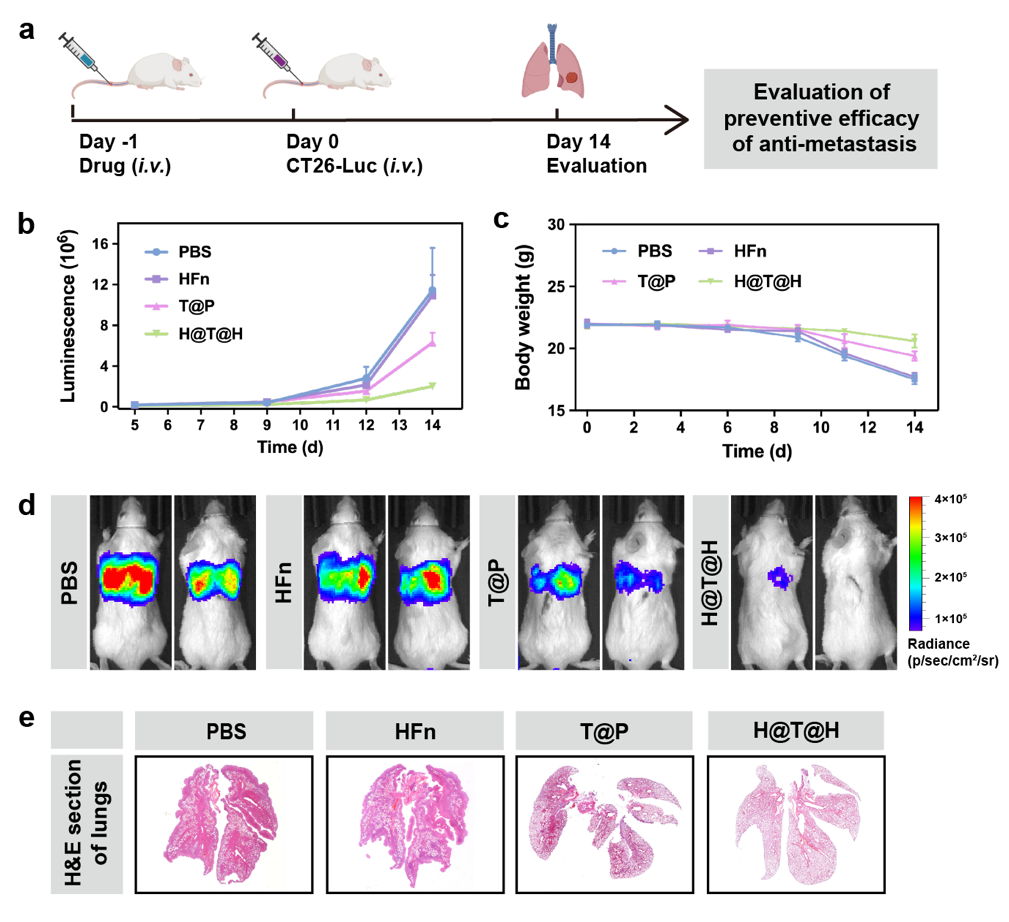


**Figure S26.** *In vivo* evaluation of anti-metastasis preventive efficacy. (a) Schematic diagram of the preventive anti-metastasis efficacy evaluation experiment. (b) Metastatic tumor growth curves of mice pretreated with a single injection of different drugs. (c) Body weight curves of mice in the different treatment groups. (d) *In vivo* bioluminescence imaging of mice bearing metastatic CT26-Luc tumors on day 14. (e) H&E-stained lung sections of mice in different treatment groups. Data are shown as means ± SEM (*n* = 5).


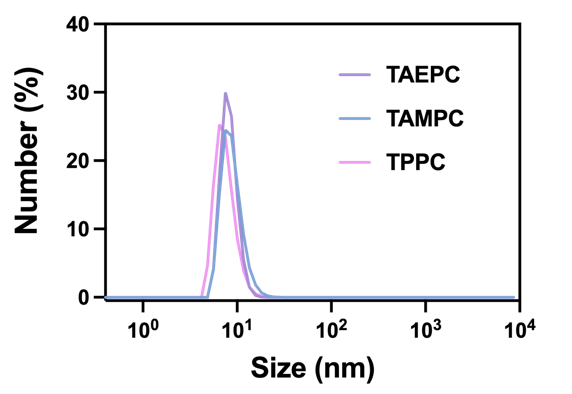


**Figure S27.** Hydrodynamic size distributions of different aminated fullerene derivatives


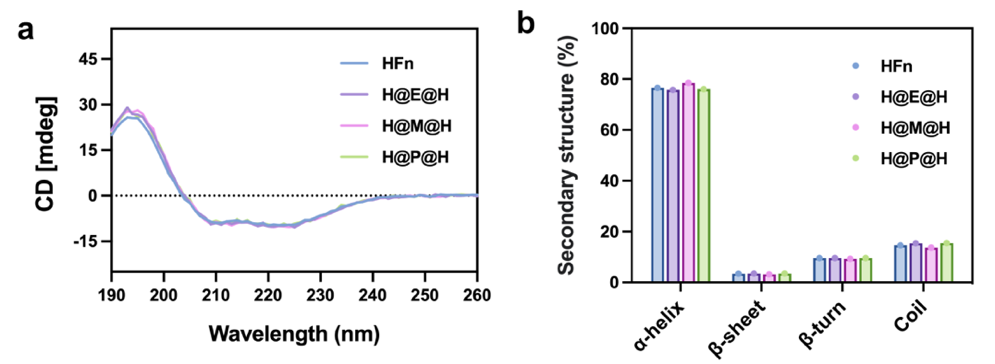


**Figure S28.** CD analysis of protein conformation. (a) CD spectra of HFn, H@E@H, H@M@H and H@P@H. (b) Quantitative analysis of the secondary structure.


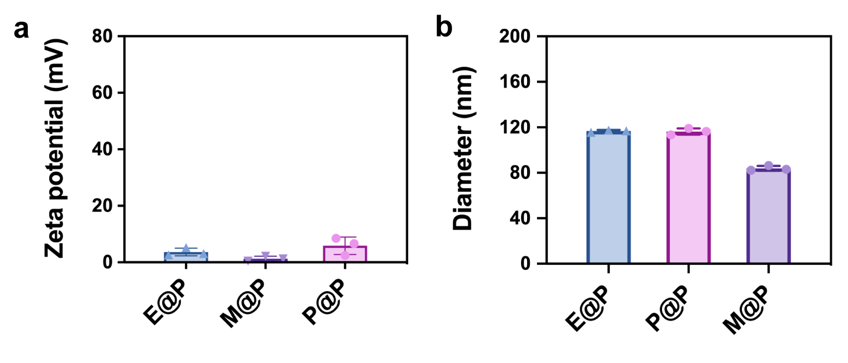


**Figure S29.** Characterization of PEG-coated aminated fullerene derivatives**.** (a-b) Zeta potential (a) and diameter (b) of E@P, M@P, and P@P. Data are shown as means ± SD (*n* = 3).

**Supplementary Tables**

**Table S1.** BLI assay of TAPC and HFn affinity

| **Sensor** | **Loading sample** | **Analyte** | ***K_D_* (M)** | ***K_on_*** **(Ms^-1^)** | ***K_dis_*** **(s^-1^)** |
| --- | --- | --- | --- | --- | --- |
| Ni-NTA | HFn with His-tag | TAPC | 7.46×10^-7^ | 2.96×10^2^ | 2.21×10^-4^ |

***K_D_:*** the affinity constant, representing the affinity. ***K_on_:*** the binding constant, representing the rate of complex formation per unit time. ***K_dis_:*** the dissociation constant, representing the percentage of dissociated complexes per unit time.

**Table S2**. Diameters and PDIs of HFn-based supramolecular assemblies of different aminated fullerenes (means ± SD, *n* = 3).

|  | **Diameter (nm)** | **PDI** |
| --- | --- | --- |
| **H@E@H** | 107.0 ± 1.1 | 0.11 ± 0.01 |
| **H@M@H** | 117.3 ± 7.3 | 0.13 ± 0.02 |
| **H@P@H** | 126.0 ± 1.3 | 0.12 ± 0.02 |

**Table S3.** Sequence of primers

| **Gene** | **Primer** | **Sequence** |
| --- | --- | --- |
| β-actin | Forward Primer  Reverse Primer | 5'-CATGTACGTTGCTATCCAGGC-3'  5'-CTCCTTAATGTCACGCACGAT-3' |
| c-Myc | Forward Primer  Reverse Primer | 5'-GTCAAGAGGCGAACACACAAC-3'  5'-TTGGACGGACAGGATGTATGC-3' |
| CCND | Forward Primer  Reverse Primer | 5'-CCTCGGTGTCCTACTTCAAATG-3'  5'-GCGGTCCAGGTAGTTCATG-3' |
| CDK4 | Forward Primer  Reverse Primer | 5'-ATGGCTACCTCTCGATATGAGC-3'  5'-CATTGGGGACTCTCACACTCT-3' |
| Vimentin | Forward Primer  Reverse Primer | 5'-AGTCCACTGAGTACCGGAGAC-3'  5'-CATTTCACGCATCTGGCGTTC-3' |
| N-cadherin | Forward Primer  Reverse Primer | 5'-AGCCAACCTTAACTGAGGAGT-3'  5'-GGCAAGTTGATTGGAGGGATG-3' |
| E-cadherin | Forward Primer  Reverse Primer | 5'-AAAGGCCCATTTCCTAAAAACCT-3'  5'-TGCGTTCTCTATCCAGAGGCT-3' |
| Snail 1 | Forward Primer  Reverse Primer | 5'-GGAAGCCTAACTACAGCGAG-3'  5'-CAGAGTCCCAGATGAGCATTG-3' |
| Snail 2 | Forward Primer  Reverse Primer | 5'-AGCATTTCAACGCCTCCA-3'  5'-GGATCTCTGGTTGTGGTATGAC-3' |

**References**

[1] Y. Lu, J. Li, X. Liu, D. He, L. Yang, W. Zhou, X. Wang, S. Chen, S. Chen, Y. Liu, X. Wang, J. Li, J. Huo, Y. Liu, Z. Wang, M. Liu, Y. Wang, Y. Li, F. Zhao, S. Li, J. Wei, J. Liu, W. Li, C. Wang, *CCS Chemistry* 2024, 6, 2392.

[2] J. Huo, J. Li, Y. Liu, L. Yang, X. Cao, C. Zhao, Y. Lu, W. Zhou, S. Li, J. Liu, J. Li, X. Li, J. Wan, R. Wen, M. Zhen, C. Wang, C. Bai, *Adv Sci* **2022**, 9, 2201541.

[3] K. Fan, X. Jia, M. Zhou, K. Wang, J. Conde, J. He, J. Tian, X. Yan, *ACS Nano* **2018**, 12, 4105.
